# Exploring boundary conditions of goal-driven attentional capture by affective categories: the role of prioritisation in working memory

**DOI:** 10.1007/s00426-025-02227-9

**Published:** 2026-02-26

**Authors:** Chris R. H. Brown

**Affiliations:** School of Psychology, Bournemouth University, Talbot Campus, Fern Barrow, Poole, BH12 5BB UK

**Keywords:** Attentional bias, Visual Working memory, Threat; Goal-driven, Action preparation

## Abstract

When stimuli are retained in visual working memory (VWM) external stimuli which overlap with this representation capture attention when performing a visual task. It has not been determined, however, whether this mechanism can partly account for attentional capture by categories of real-world affective stimuli. Across five dual-task visual search and VWM change detection experiments (4/5 pre-registered; total *N* = 119) participants had to detect the change in either positive (kitten) or threat-related (spider) animal exemplars, whilst performing an intervening visual search task with peripheral distractors from these affective categories. The affective stimulus associations were confirmed by arousal and valence ratings in all five samples and in an independent sample (*n* = 82). It was hypothesised that threat-related and positive distractors would capture attention more, versus a neutral (bird or no distractor) baseline, when matching the contents of VWM. Experiments 1–3, however, found no evidence of increased capture by VWM-matching affective stimuli, though there was cumulative evidence of goal-independent capture by threat-related distractors. When, however, the trial structure became unpredictable, requiring constant preparation for the VWM task response (Experiment 4), or advanced action preparation to the VWM task was enabled (Experiment 5), then VWM-matching threat-related distractors caused greater attentional capture. This VWM-driven capture, however, was not found for positive distractors in any experiments. The results probe the boundary conditions when VWM contents drive attentional capture by entirely task-irrelevant affective categories, and suggests that background memory representations may not influence attention unconditionally, and instead may depend partly on their current prioritisation.

**Supplementary Information:**

The online version contains supplementary material available at 10.1007/s00426-025-02227-9.

## Introduction 

In everyday life our attention system must perform a balancing act by intentionally prioritising stimuli relevant to our current goals, whilst also flexibly shifting attention to stimuli which are not currently relevant but could signal potential danger or reward. Evidence has shown that threat-related stimuli which signal potential aversive outcomes capture our attention, even when they are presented as task-irrelevant distractors and are no more physically salient than other objects. This has been shown across a range of visual stimuli such as features predicting aversive outcomes, dangerous animals and objects, and emotional faces associated with danger such as fear or anger (Devue et al., 2011; Eimer & Kiss, 2007; Hodsoll et al., 2011; Lipp & Derakshan, 2005; Most et al., 2005; Schmidt et al., 2015).

Contemporary theories of attention have suggested that this effect is due to a specific value-driven mechanism which biases selection independent of an individual’s current goals and intentions, as well as the physical features of the stimulus, such as brightness or colour (Anderson et al., 2021; Awh et al., 2012; Theeuwes, 2019; Vuilleumier, 2005). With evidence demonstrating that attention is biased towards non-salient stimuli despite awareness of their task-irrelevance and their counterproductive effect on current task performance, consistent with the proposed goal-independent mechanism (Anderson & Britton, 2020; Mikhael et al., 2021).

Whilst this line of questioning has produced compelling findings outlining the limits of human attentional control, the focus on purely the value-driven mechanism of attention may have overshadowed the investigation of other mechanisms of attentional capture. It may be that there are other plausible mechanisms which drive distraction by affective stimuli in different contexts, in addition to value-driven effects. Indeed, evidence has shown that involuntary attentional capture by task-irrelevant stimuli can also be paradoxically caused by congruence with our goals, as the instruction to detect specific features or a category of objects is sufficient to cause involuntary orienting of attention to peripheral stimuli which match the features of the target (Folk et al., 1992, 2002; Wyble et al., 2013; see Busel et al., 2020 for review). The current investigation therefore explores whether our current top-down goals contribute to the distraction that we experience from affective stimuli, independent of purely their value associations.

In support of this hypothesis, evidence has shown that when individuals adopt a top-down goal to detect a category of affective stimuli (e.g., fearful and angry faces, dangerous animals, scenes of injury) then task-irrelevant distractors from that category capture attention more strongly than when incongruent with this goal (Brown, Forster & Duka., 2018; Brown, Berggren & Forster., 2020a; 2020b; Victeur et al., 2019). This goal-driven involuntary attentional capture appears to generalise to all exemplars from that affective category, rather than purely the specific features of the target, thus suggesting a plausible alternative mechanism driving elevated attentional capture by affective stimuli.

It should be noted that whilst this shows that goal-driven processes can contribute to attentional capture by affective stimuli, these earlier tasks may not be reflective of the conditions in which we tune attention to an affective category in the real-world. Within these task designs the individual holds a single goal to detect a threat-related category. In a real-world situation, this would likely only be implemented in unambiguously threatening situation, when threat-detection’s priority completely surpasses all other possible goals (e.g., looking for attackers when escaping an ongoing violent attack). Often the threats we perceive in everyday life are actually more ambiguous, and require vigilance for threat alongside performance of a primary goal (e.g., looking out for potential attackers in an unfamiliar carpark at night, whilst searching for our car).

Empirical evidence has supported the idea that we are capable of maintaining multiple goals active at one time to achieve a range of desired outcomes, and either pursue these goals concurrently or switch between them in sequence (for reviews see Kung & Scholer, 2020; Orehek & Vazeou-Nieuwenhuis, 2013). Indeed, evidence from spontaneous task-switching experiments has shown that we do not often pursue one single goal for extended periods of time, and when given the option we voluntarily switch between goals; especially if switching to another goal is expected to result in a more valued outcome (Kessler et al., 2009; Frober & Dreisbach, 2016; 2017). A real-world example of this concurrent goal pursuit could be giving a presentation, when we may attend to the slides on a screen and what we are about to say, whilst also tuning attention to the facial expressions of the audience; or when clearing out a storage cupboard we may be looking for an object whilst also being vigilant for a spider that we would want to avoid.

Consistent with this ability to maintain multiple goals active at once, research utilising dual-task designs have consistently demonstrated that whilst performing a visual search task individuals are able to represent other visual stimuli active in visual working memory (VWM), despite these being relevant to a distinct visual task (Downing et al., 2000; Woodman et al., 2001). Interestingly, evidence suggests that any features which match those held as a representation in VWM involuntarily capture attention despite being task-irrelevant, resulting in slower target detection in a concurrent task (Bahle et al., 2020; Beck et al., 2012; Downing., 2000; Olivers et al., 2006; Soto et al., 2005). Further, it has been shown that this effect also occurs for real-world objects, and appears to generalise along a semantic dimension, as stimuli which are from the same category as the target object stored in VWM also appear to disrupt visual search (Balani et al., 2010; Calleja & Willoughby, 2023; Soto & Humphreys, 2007).

Given the evolutionary importance of avoiding danger (Boyer & Bergstrom, 2011), it is plausible that we may regularly prioritise affective information in working memory, especially threat-related stimuli, and that this could drive an involuntary bias through this top-down mechanism. Consistent with this theory, evidence does suggests that we frequently engage in spontaneous thoughts regarding motivationally relevant topics, especially our current concerns and stressors (Klinger et al., 1980; Killingsworth & Gilbert, 2010); and VWM research, specifically, shows that when emotional stimuli are maintained in VWM they are given greater priority over equally relevant neutral stimuli, as demonstrated by more precise and accurate recall (Jackson et al., 2008, 2014; Lee & Cho, 2019; Thomas et al., 2014).

The current investigation will therefore explore how maintaining affective stimuli in VWM as a background goal, concurrent to a visual search task, influences involuntary attentional capture by task-irrelevant but VWM-matching affective stimuli. If this hypothesised VWM-driven capture is found, then it would highlight the role of potential top-down drivers of attentional biases to emotion observed in other contexts.

### Guidance by affective background goals

The few existing studies which have explored VWM-driven affective biases have yet to show clear evidence that retaining an affective stimulus in VWM causes a generalised involuntary attentional capture by all exemplars from the affective category. Instead, the current experimental evidence is more inline with participants attending the stimuli strategically in order to refresh the memory of the stimulus in VWM, to aid later recall (Woodman & Luck, 2007). For instance, Yao et al. (2019) found that maintaining an angry face image in VWM only resulted in an increased attentional capture by angry faces which were identical to the VWM cue, and that other angry faces failed to capture attention more in this condition.

Further, parallel to threat-related distraction research, evidence with motivationally relevant reward associated categories has shown a similar lack of generalisation. For instance, Brown et al. (2018) found no evidence that retaining an alcohol image in VWM increased attentional capture by visually similar but non-identical peripheral alcohol distractors during a rapid serial visual presentation task. Moreover, the instances in which VWM-matching reward associated categories, such as food stimuli, do capture attention it has been with stimuli that are identical to the image in VWM (Higgs et al., 2012; Kumar et al., 2016). Thus, the majority of findings show a lack of evidence of a general bias to non-identical stimuli, which is more consistent with a strategic allocation of attention to aid VWM task performance, rather than involuntary capture.

There is, however, one study which has shown evidence that a background goal can induce a broad attentional capture by a category of affective stimuli. In this task, Vogt et al. (2022) instructed participants to detect randomly appearing angry faces, whilst also completing a primary dot probe task. This resulted in greater attentional capture by task-irrelevant angry faces in the dot-probe task, relative to when the goal was for a non-affective category. Though the results are in line with a background goal-driven capture, the angry faces were presented prior to the primary target and in potential target locations where participants’ attention would already be allocated. This would reduce the motivation and ability to inhibit the distractor, as suppression of the distractor and its location would likely disrupt detection of a subsequent target when it appeared in the same location (Ferrante et al., 2023). To fully conclude that the attentional capture caused by a background goal in VWM was completely involuntary, the conditions that support effective inhibition are required. Specifically, the task-irrelevant capture must occur either when the distractor is in an entirely irrelevant location (Forster & Lavie, 2008), or if the distractor reliably appears concurrent to the target, thus allowing it to be easily discriminated and inhibited (Gaspelin et al., 2015). Whilst the current limited evidence supports the hypothesis that a background VWM representation can induce attentional capture by affective categories, these findings have been tested in contexts in which are most sensitive to processing of these stimuli.

### Current investigation

To explore whether maintaining an affective stimulus in VWM can induce early attentional capture by entirely task-irrelevant affective distractors, in competition with a primary visual target, a VWM change detection task was combined with a visual search task (adapted from Forster & Lavie, 2008). Within this task, the affective distractors were presented as brief (i.e., 100ms) peripheral distractors alongside the target stimuli in entirely task-irrelevant locations, which should facilitate distractor inhibition and target engagement. Further, at this brief presentation time and irrelevant location, any interference would be reflective of involuntary attentional capture, and less reflective of delayed disengagement or strategic attentional deployment.

To further prevent strategic use of the distractor to refresh the VWM representation, the specific distractor stimulus presented as a peripheral distractor was always a different exemplar from that retained in VWM, even when from the same category. Thus, slower target detection when the distractor is presented should be reflective of entirely involuntary capture, independent of spatial attention or strategic processing, and in direct competition with currently task-relevant stimuli.

The affective stimuli selected for the current task were animals with clear affective associations. Specifically, spiders were selected as threat-related stimuli, birds as a neutral baseline stimulus, and kittens as a comparative positive category stimulus. These categories have been widely used in investigations of attentional bias to affective stimuli, with ratings of these stimuli confirming their affective status (Abado et al., 2023, 2024; Brown et al., 2020a; Gerdes et al., 2008; Vromen et al., 2015, 2016). The selection of spiders as threat-related stimuli is especially relevant, as it is hypothesised that spiders would be one of the earliest threats posed to our direct ancestors, and that detection of these features would confer an evolutionary advantage (Ohman, 1986).

Empirical evidence is consistent with an evolved prioritisation of spider threat in attention, as it has been found that the aversive association with spider stimuli and their prioritisation in attention is either innate or rapidly develops during infancy (Hoehl et al., 2017; LoBue, 2010; Rakinson & Derringer, 2008). Further, a consistent attentional bias to task-irrelevant spiders has also been found in adults across a range of investigations and tasks (Basanovic et al., 2017; Brosch & Sharma, 2005; New & German, 2015; Rinck et al., 2006). The selection of spiders as an affective threat-related category is therefore ideal for the current investigation.

It was expected that the current paradigm would reveal that maintaining an exemplar from either a positive or threat-related category of animals (i.e., kittens or spiders) in VWM as a background visual goal, prior to a visual search task, would result in greater capture from VWM-matching peripheral distractors from that category during visual search (as reflected by slower target detection in the presence of a distractor versus neutral baseline), relative to when an exemplar from the other affective category was maintained in VWM. Though the primary focus of the current study was attentional capture by threat-related stimuli (i.e., spiders), it was also expected that positive stimuli (i.e., kittens) would show a similar pattern of elevated attentional capture when congruent with the active representation in VWM.

## Experiment 1

### Methods

#### Participants

The sample sizes for all studies were designed to be sufficiently large to detect specific interaction contrast, which was the difference in reaction time between neutral and threat-related distractors, when compared between VWM-matching and mismatching conditions. This contrast could be isolated through a pairwise comparison of the difference between neutral versus threat-related distractor reaction times between VWM-matching and mismatching condition.

Within the literature there are very few studies exploring VWM-driven attentional capture by complex real-world images in peripheral locations. The most similar study to the current design was therefore that of Tan et al. (2015), who used a near identical letter visual search task with peripherally located distractors, which could either match or mismatch the contents of VWM. The effect reflecting the difference between matching and non-matching distractors was *d*_*z*_ = 0.88.^1^ Though this was a main effect contrast, it was expected that when all distractors mismatched the contents of VWM there would be a near zero difference (based on recent findings Brown et al., 2020a; Vromen et al., 2016), meaning the interaction would be similar to the main effect difference. Used this effect to compute an *a priori* power analysis using G*Power software (Faul et al., 2009) suggested a minimum sample of 16 participants would be required (α = 0.05, 1-β = 0.9, two-tailed).^2^

However, to account for potentially smaller effects in the interaction, a larger total sample of 24 participants was recruited. This was selected based on previous sample sizes utilised to detect within-participants goal-driven capture by threat-related animals (Brown et al., 2020a; Vromen et al., 2016). This sample size was retained across all subsequent experiments to facilitate comparison across samples. A sensitivity power analysis indicated that the final sample was sufficient to detect effects as small as *d*_*z*_ = 0.69 (α = 0.05, 1-β = 0.9, two-tailed).

In Experiment 1, this initial sample of 24 participants was recruited from the student participant pool in exchange for course credits, though two were excluded for poor performance on the task (< 0.60 accuracy on either letter identification or VWM change detection for either animal). Due to disruption from COVID-19 lockdowns, these two participants weren’t replaced. The final sample of 22 participants consisted of 5 men and 17 women, with a mean age of 27.45 years (*SD* = 6.17). Twenty of the 22 participants were right-handed. The average level of trait anxiety was 44.10, *SD* = 11.47, state anxiety was 37.86, *SD* = 8.14, and average spider fear score was 2.93, *SD* = 1.81. These were measured with the State-Trait Anxiety Inventory and Spider Fear Questionnaire (Spielberger et al., 1983; Szymanski & O’Donohue, 1995), see below for further details.

All studies across the investigation were approved by departmental ethics (ethics code: CB_ERP1_21), and recruitment was conducted between the years 2019 and 2023.

#### Stimuli and materials

##### Concurrent VWM Change Detection and Visual Search Task.

**Fig. 1:**
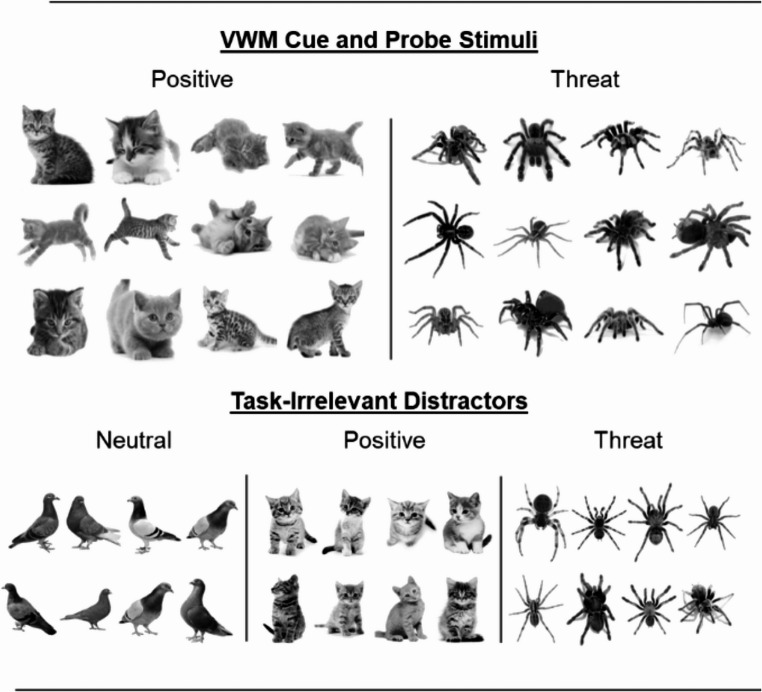
All VWM cue and probe, and distractor stimuli across threat-related, positive, and neutral categories, along with the affective rating task

**Fig. 2:**
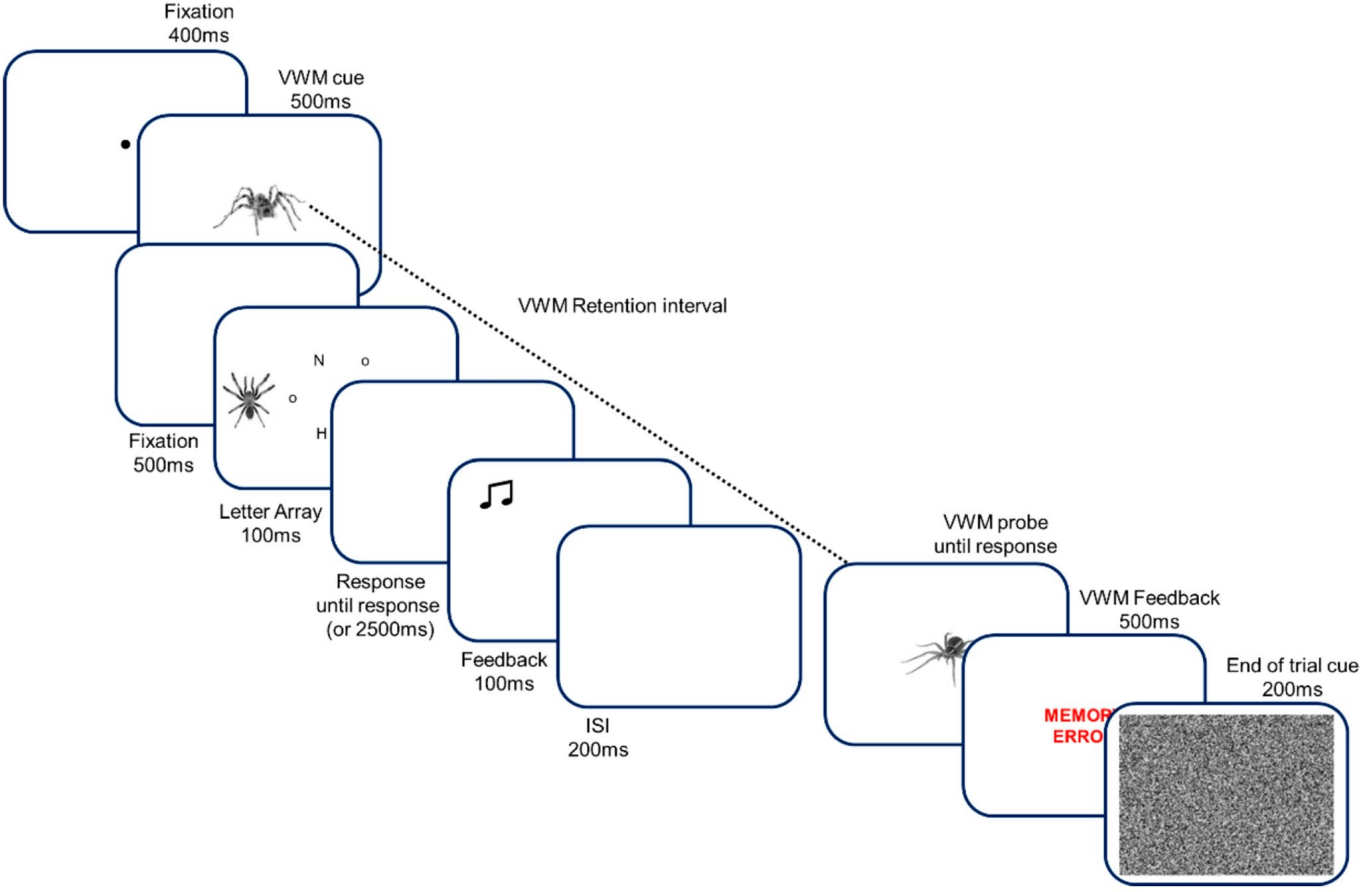
A diagram of trial sequence for combined Visual Working Memory (VWM) change detection task and visual search task with irrelevant peripheral distractor – with the threat-related stimulus condition for both, and error feedback on both tasks

 See Figs. 1 and 2 for depiction of all stimuli and task structure. The stimuli were presented using E-prime 2.0 software on a 24-inch Dell monitor with a screen resolution of 1920 × 1080 and a refresh rate of 60 Hz. Participants viewed the monitor from a distance of 60 cm. The design of the experiment was a 2 × 3 repeated measures design, with the type of VWM cue varied across block (i.e., positive VWM cue, threat VWM cue), and type of distractor varied across trials within each block in a random order (i.e., neutral bird distractor, threat-related spider distractor, positive kitten distractor). In total there were four blocks of 72 trials, resulting in 288 trials in total across the 6 conditions (48 trials per condition).

All positive, threat-related, and neutral animal images presented as both VWM cue and probe, and distractor images, were sourced from Google Images from non-copyrighted sources (see Fig. 1). They were all converted to grayscale to remove salient colours and were converted to an approximately equal size across animals. All background colour was removed from the images, and replaced with a white background. VWM stimuli were selected to have a range of orientations and sizes to encourage participants to represent all exemplar features, rather than a single feature across trials.

The category of affective animal in the change detection task remained consistent across a block, with the animal type interleaved across blocks (e.g., Kitten – Spider – Kitten – Spider), and the order counterbalanced between participants. In total there were 12 kitten stimuli and 12 spider stimuli which were presented as VWM cue or probe stimuli, with the selection taken randomly from this pool of images. Each VWM cue stimulus was presented an equal number of times within each distractor condition. The VWM cue stimuli were resized to fit within a 3.72° × 3.72° square, though the outline of the stimulus varied within this area.

The distractor images included greyscale images of 8 positive kitten stimuli, 8 threat-related spider stimuli, and 8 neutral bird stimuli. All of these were resized to fit within a 2.31° × 3.72° vertical rectangle, which could appear to the left or the right of the letter array at an eccentricity of 3.15° from the central position. It appeared an equal number of times to the left or right in each condition, with the opposite peripheral position to the distractor left blank. Exemplars were randomly selected each trial, but were presented an equal number of times within each VWM type condition. All distractors were vertically aligned to fit in the peripheral location and ensure the different distractor types were approximately equal size.

The visual search letter array and distractors were presented for only 100ms, in order to assess the influence of early attentional capture by affective stimuli, rather than potentially later effects on attention. After the distractor and target array, there was a response window in which participants identified whether an X or N target letter was present, responding with the ‘2’ key for the N, and ‘0’ key for the X on the number line using the left and right hands, respectively. The response window continued until either 2500ms had passed, or a response was registered. After the response window, a 100ms feedback screen was presented with a beep on error trials.

After a 200ms ISI, a VWM probe exemplar was presented in the centre of the screen to the same dimensions as the VWM cue. On half the trials within each condition the probe was identical to the VWM cue, whilst on the other half it was replaced with a randomly selected exemplar from the same category. Participants responded using the ‘s’ and ‘d’ keys to signal same and different, respectively. After the VWM response, participants were presented with visual feedback for 500ms, which consisted of the text “MEMORY CORRECT!” in blue, or “MEMORY ERROR” in red. At the end of each trial, a 18.72° × 14.06° white noise image was presented to fill the area of the previous stimuli to signal the end of the trial.

Target position, target type, distractor position, distractor type, and VWM change were all counterbalanced within each condition, within each block. The order of the trials, position and identity of the filler letter in the visual search array were randomly selected each trial. All distractor and VWM images were randomly selected each trial, but appeared an equal number of times within each condition.

Prior to the task participants completed a practice block, which consisted of 12 example trials where stimuli remained on screen until response, and 24 full speed practice trials. This was identical to the main blocks, but the distractors were always grey rectangles, and the VWM cues and probes were drawn from 12 grey-scaled goldfish exemplars.

##### Self-Assessment Manikin Affective Dimension Ratings** (**Bradley & Lang, 1994**).**

Participants completed three rating trials, one for spiders, one for kittens, and one for bird distractor stimuli. On each trial participants were presented with all 12 stimuli to be presented as distractors at once, in a 2 × 6 grid. On each trial participants were instructed to rate the group of images as a whole, rather than specific exemplars, on their valence and arousal which they elicited.

Ratings were along a 1–9 scale, for valence, ratings were between the labels, “Extremely unpleasant” to “Extremely pleasant”, with the mid-point labelled as “Neutral”. For arousal, the ratings were between the labels of “Calm/bored” to “Excited/agitated”. The order that the animals were rated on their valence and arousal was randomised. Each scale was accompanied by self-assessment manikins to assist the ratings (Bradley & Lang, 1994). These were simple pictorial representations of the valence and physiological arousal states one may feel whilst viewing the images which acted as a reference point.

##### State-Trait anxiety inventory (STAI; Spielberger et al., 1983). 

The STAI includes two 20 item scales which measure current state anxiety and general trait anxiety. Responses were along a 4-point Likert scale, ranging from *“Almost never”* to *“Almost always”* for state anxiety, and from *“Not at all”* to “*Very much so ”* for trait anxiety. The summed score for the first 20-items constituted the state anxiety outcome measure, and the summed score for the second 20-items made-up the trait anxiety outcome measure.

##### Fear of Spiders Questionnaire (FSQ; Szymanski & O’Donohue, 1995**).**

The FSQ includes 18 items measuring spider fear. Responses were along a 7-point Likert scale ranging from “*Almost never true”* to “*Almost always true”*. Within Experiment 1, a 17-item version of the FSQ was used due to a missing item during coding. To allow comparison of the FSQ score across samples, the average score was utilised as the outcome measure.

#### Procedure

After providing informed consent, participants were given full instructions and completed the practice block under supervision from the experimenter. Participants were encouraged to prioritise fast but accurate responses to the visual search task, and only accurate responses to the VWM change detection task. Participants were made aware that the distractor exemplars would always be different from the VWM exemplars to discourage participants from strategically attending to the distractors as a memory aid.

They then completed the main experimental task, before completing the self-assessment manikin affective rating task, STAI and FSQ in a random order, before being debriefed. The experiment took place in a dimly lit single participant testing cubicle.

#### Transparency and openness

All data and analyses scripts for the current investigation are available via the Open Science Framework (OSF link: https://osf.io/2ctjp/). Four of the experimental studies (Experiment 2, 3, 4 and 5) were pre-registered, also via the OSF, and these links are available in the relevant methods sections for these experiments. These pre-registrations encompass recruitment and sample size determination, methods and design, analyses, and interpretation criteria. Deviations from the pre-registration are noted in the text, and those analyses not pre-registered are labelled as exploratory.

#### Statistical analysis

The primary dependent variable for the current investigation was the reaction time (RT) to correctly respond to the target letter in the visual search task. Thus, trials in which responses were incorrect or were over 2,500ms or under 100ms were excluded. Accuracy (scored as probability ranging between 0 and 1) was analysed as a secondary dependent variable.

Given that the distractor was always different from the VWM-cue and probe, attending to it would never facilitate performance on the VWM task; however, recent evidence has found that when retaining a stimulus in VWM, external distractors which are visually similar to the contents of VWM disrupt this representation more than dissimilar distractors (Zhang & Peacock, 2025). Therefore, both correct and incorrect VWM change detection task trials were retained, as removing incorrect VWM change detection trials would have systematically excluded more trials in the VWM-matching conditions where the distractor captured attention, which would have obscured the hypothesised effect. Indeed, evidence for this selective interference by visually similar distractors was found in Experiments 2 and 4, where spider distractors selectively disrupted spider VWM change detection.

Across studies, the main aim of the statistical analysis was to assess the influence of the different affective distractor types on visual search performance when they matched/mismatched the contents of VWM. To target this specific interaction, a Bayesian pair-wise comparison was utilised, with a specific informed prior taken from published work.

For this comparison, a specific reaction time Affective Distractor Interference (ADI_RT_) score was computed to isolate the disruption to search efficiency from the affective content of the distractor. This was computed both types of affective distractor separately (i.e., positive/threat-related), and was computed by subtracting the visual search reaction time when the distractor was the neutral bird from when the distractor was the positive kitten or threat-related spider distractor. Each ADI score was computed for each participant individually. A secondary accuracy-based ADI score was also computed (ADI_accuracy_), by subtracting the accuracy when the distractor was positive or threat-related from when it was neutral. For both these ADI scores, a higher value would indicate increased interference from the affective content of the peripheral distractor. The specific test to assess the interaction between affective distractor interference was, therefore, to assess whether the ADI score was higher when it was VWM-matching versus VWM-mismatching.

The Bayesian priors utilised to assess whether the observed difference from this comparison favoured the experimental hypothesis or the null hypothesis were taken from previous published research. When comparing the reaction time-based ADI score, the prior was set to 28ms, which was taken from the VWM-matching distractor interference score taken from Tan et al. (2015), who used a nearly identical task with abstract shape VWM stimuli and distractors. This prior was set as a directional half-normal distribution, with the mean expected effect size modelled as a standard deviation centred on zero, reflecting the null. The use of an informed half-normal distribution assumes that small effects close to the null are more probable, making the analysis less sensitive to small observed effects and more sensitive to effects which align with the larger effects set as the prior. If Bayes factors, therefore, show substantial evidence favouring the experimental hypothesis, then this shows stronger evidence for the proposed theory (Dienes, 2014).

Bayes factors were computed from the analysis, and were interpreted in line with classic interpretation guidelines of evidence to assist reporting and interpretation (Jeffreys, 1961; Lee & Wagenmakers, 2014): A value close to 1 suggests that any difference is anecdotal and is inconclusive until more data are collected, whilst values greater than 3 reflect moderate evidence for the experimental hypothesis (i.e., over three times more likely under the experiment hypothesis), and less than 0.33 reflect moderate evidence for the null hypothesis (i.e., over three times more likely under the null hypothesis). It should be noted, however, the Bayes factors are interpreted as a continuous measure of evidence, rather than using strict cut-off criteria as with p-values.

Bayes factors are reported using the convention outlined by Dienes (2014), where B_H[0,28]_ denotes a Bayes factor (B) computed with a half-normal distribution (H), a distribution mode of zero reflecting the null, and a distribution of 28 reflecting the expected 28ms effect under the experimental hypothesis. In analyses where a uniform distribution was applied due to lack of prior evidence, this was denoted with B_U_ rather than B_H_.

For the accuracy ADI score, previous effects from similar irrelevant distractor tasks were often extremely small (e.g., 0.03 distractor cost; Forster & Lavie, 2008), due to RT often being the prioritised measure. Setting these would therefore be not sufficiently informative, thus a prior effect of 0.12 was set based on earlier work using a different task, which compared the difference between goal-matching and mismatching affective versus neutral animals in an accuracy-based task (Brown et al., 2020a). Due to the prior being taken from a different task, there was greater uncertainty about the magnitude of the effect. This prior was therefore modelled as a uniform distribution with a lower end set to zero reflecting the null result, and the upper limit set to the 0.12 prior. The uniform distribution is agnostic to the probability of the effects, and results in Bayes factors which are more data driven and less influenced by the prior expectations of the effect size (Dienes, 2014).

To explore the effects of distractor regardless of whether they matched/mismatched the contents of VWM, the contrast between the neutral distractor and affective distractors were made. These utilised the same Bayesian priors from the VWM-match versus VWM-mismatch capture effect, as based on previous findings it was expected that the VWM-mismatch condition would result in near zero ADI_RT_/ADI_accuracy_ scores (e.g., Brown et al., 2020a; Vromen et al., 2016). For follow-up exploratory analysis, where no clear prediction about the direction or magnitude of the effect was made, two-tailed pairwise t-tests and repeated measures ANOVAs were applied. It should be noted that repeated measures ANOVAs and t-tests were pre-registered in Experiments 2–5 as part of the main analysis, alongside Bayes factors. To avoid the redundancy from reporting multiple inferential statistics, and the possible ambiguity from conflicting frequentist and Bayesian analyses (Dienes, 2024), only Bayes factors are reported in the main manuscript; however, full frequentist statistics analysis of the data is reported in Supplementary materials 1.

The primary interest in the current investigation was the influence of the content of VWM on distractor interference, however, to explore how performance differed across the two VWM types, and whether the distractor disrupted this performance, a repeated measures ANOVA was conducted on the VWM change detection accuracy.

## Discussion and results

**Fig. 3:**
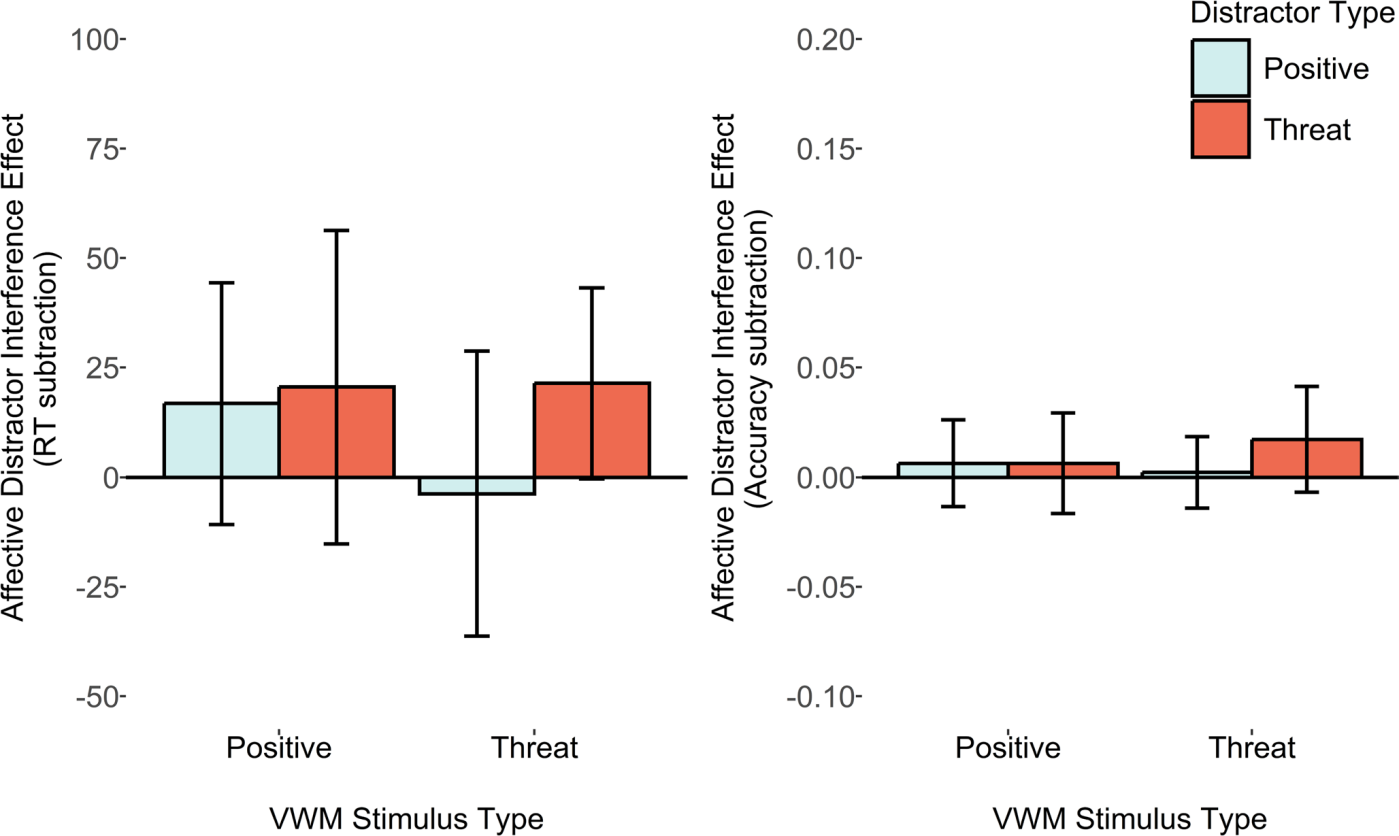
Plots depicting average reaction time and accuracy affective distractor interference scores (relative to a neutral distractor condition). Error bars represent within-subjects 95% confidence intervals

The key *a priori* pairwise comparison of the ADI_RT_ score (see Fig. 3) between VWM-match versus mismatch conditions revealed only a minimal increase in distractor interference in the VWM matching condition, with evidence anecdotally favouring the null. This was true for the threat-related distractor, both when analysing the reaction time ADI_RT_ score, *M*_*diff*_ = 1ms, *SD*_*diff*_ = 97, *d*_*z*_ = 0.01, B_H[0, 28]_ = 0.61, and the accuracy ADI_accuracy_ score between VWM matching and mismatching conditions, *M*_*diff*_ = 0.01, SD = 0.08, *d*_*z*_ = 0.13, B_U[0, 0.12]_ = 0.33. A similar pattern was observed for the positive distractor, for the ADI_RT_ score, *M*_*diff*_ = 20ms, *SD*_*diff*_ = 99, *d*_*z*_ = 0.21, B_H[0, 28]_ = 1.27, and the ADI_accuracy_ score between VWM matching and mismatching conditions, *M*_*diff*_ < 0.01, *SD*_diff_ = 0.06, *d*_*z*_ = 0.01, B_U[0, 0.12]_ = 0.18.

Assessing the follow-up comparisons for each individual ADI_RT_ score (see Table 1), to test whether there was slower and more inaccurate responding for affective distractors relative to neutral distractors in both conditions, revealed that for the threat-related distractors there was anecdotal evidence favouring the experimental hypothesis within the VWM-mismatching condition, *d*_*z*_ = 0.25, B_H[0, 28]_ = 1.45, and the matching condition, *d*_*z*_ = 0.35, B_H[0, 28]_ = 2.38. Assessing the pattern with the ADI_accuracy_ score as the secondary dependent variable revealed evidence anecdotally favouring the null hypothesis in both VWM-matching, *d*_*z*_ = 0.29, B_U[0, 0.12]_ = 0.54, and moderately favouring the null for mismatching conditions, *d*_*z*_ = 0.13, B_U[0, 0.12]_ = 0.19.

**Table 1:** Means and standard deviations (*SD*) for reaction time (RT) and accuracy (probability), and affective distractor interference (ADI) scores, as well as accuracy in the change detection task. Bayes factors are reported with priors listed in notation. Bayes factors showing substantial evidence for the experimental hypothesis (i.e., BF > 3) are highlighted in bold

	VWM	Distractor	RT (SD)	ADI_RT_ score	B_H[0, 28]_	Accuracy (SD)	ADI_accuracy_ score	B_U[0, 0.12]_	VWM (SD)
Experiment 1 (*n* = 22)	Positive	Neutral	934 (176)	-	-	0.83 (0.08)	-	-	0.83 (0.08)
		Positive	950 (168)	17 (66)	1.36	0.80 (0.08)	0.01 (0.04)	0.19	0.79 (0.08)
		Threat	954 (194)	20 (83)	1.45	0.81 (0.10)	0.01 (0.05)	0.19	0.81 (0.10)
	Threat	Neutral	906 (173)	-		0.82 (0.11)	-	-	0.82 (0.11)
		Positive	903 (160)	−4 (76)	0.43	0.79 (0.12)	0 (0.05)	0.13	0.79 (0.12)
		Threat	928 (187)	21 (61)	2.38	0.80 (0.10)	0.02 (0.07)	0.54	0.80 (0.10)

For the positive distractors there was only anecdotal evidence of reaction time distractor interference in the VWM matching, *d*_*z*_ = 0.25, B_H[0, 28]_ = 1.36, and anecdotal evidence favouring the null hypothesis in the VWM-mismatching condition, *d*_*z*_ = − 0.05, B_H[0, 28]_ = 0.43. Reanalysis with accuracy as the dependent variable revealed Bayes factors which moderately favoured the null hypothesis for positive distractors, both within the VWM-matching condition, *d*_*z*_ = 0.14, B_U[0, 0.12]_ = 0.19, and VWM-mismatching condition, *d*_*z*_ = 0.05, B_U[0, 0.12]_ = 0.13.

### VWM performance

When assessing the accuracy of the VWM change detection accuracy across the three distractor types in the kitten and spider VWM conditions, there was no significant main effect for VWM type, *F*(1,21) = 0.19, *p* =.669, *ƞ*^*2*^_*p*_ = 0.01, distractor type, *F*(2,42) = 2.79, *p* =.073, *ƞ*^*2*^_*p*_ = 0.18, or their interaction, *F*(2,42) = 0.06, *p* =.946, *ƞ*^*2*^_*p*_ < 0.01.

Based on the behavioural findings of Experiment 1 it cannot be concluded that background goals to respond to affective stimuli stored in VWM can drive involuntary capture in the current task, as the Bayes factors showed inconclusive evidence. One potential issue could be that the stimuli used as distractors were not seen as sufficiently affective, and this is why the distractors failed to capture attention even when stored in VWM.

### Meta-analysis of affective ratings across experiments

Across all samples within the current investigation (*k* = 5; total *N* = 119), participants provided valence and arousal ratings for the distractor stimuli (see Experiment 1 methods for full details). To determine whether the stimuli were perceived as sufficiently affective and emotionally arousing these ratings were analysed using an exploratory fixed effects meta-analysis, computed with the R *metafor* package (Viechtbauer, 2010). This allowed both the assessment of the cumulative ratings, as well as whether there was significant heterogeneity in the ratings across studies.

For valence and arousal ratings, the overall ratings were significantly different across distractor types (see Table 2). With the positive kitten distractors being significantly more pleasant and emotionally arousing versus the neutral bird distractor. The threat-related spider distractors were also significantly more unpleasant and more emotionally arousing than the neutral bird distractor. There was no significant evidence of heterogeneity in valence ratings, *Q*(5) = 8.60, *p >*.072, or arousal ratings, *Q*(5) = 7.42, *p* >.115, across all samples, indicating a relatively consistent judgment across studies, making behavioural variation across studies unlikely to be due to differences in the perceived affective content of the distractors.

### Affective ratings in independent sample

The affective ratings were taken at the end of each experiment, allowing direct measures of relevant participants’ perceptions. These could, however, have been influenced by the exposure in the experiment. To confirm that the differences in ratings didn’t only appear after repeated exposure in the task (Montoya et al., 2017), an independent sample (*N* = 82) of participants was recruited to assess the pattern of valence and arousal ratings of the distractor images (see Supplementary materials 2 for sample details). The results (see Table 2) revealed the same general pattern of ratings as participants from within the Experimental samples, confirming that the different perceived affective associations weren’t due only to exposure during the task.

**Table 2:** Mean and standard deviation (SD) ratings from the self-assessment manikin along dimensions of Valence and arousal for all peripheral distractor stimuli. Effect sizes and significance values from two-tailed t-tests are reported for the comparison versus neutral bird distractor stimuli

		Mean Valence Rating (SD)	Cohen’s d_z_	*p*-value	Mean Arousal Ratings (SD)	Cohen’s d_z_	*p*-value
Cumulative effects from Experiments 1–5 (*N* = 119)	Neutral birds	5.17 (1.35)	-	-	2.74 (1.82)	-	-
	Positive kittens	7.86 (1.45)	1.34	< 0.001	3.82 (2.64)	0.33	< 0.001
	Threat spiders	3.44 (1.52)	− 0.85	< 0.001	4.29 (2.48)	0.49	< 0.001
Independent sample (*n* = 82)	Neutral birds	3.66 (2)	-	-	2.90 (1.99)	-	-
	Positive kittens	6.93 (2.23)	1.03	0.001	4.63 (2.70)	0.52	0.001
	Threat spiders	2.90 (2.52)	− 0.26	0.024	4.02 (3.02)	0.36	0.002

Emotionally arousing stimuli have been found to bias attention (Vogt et al., 2008) and been linked to prioritisation of stimulus features in VWM (Lee & Cho, 2019). Therefore, the lack of substantial increase in capture by affective categories stored in VWM is unlikely to be due to the stimuli lacking affective associations. Further, the ratings of these stimuli did not significantly deviate across the current series of Experiments, indicating that perceptions of the current stimulus set were consistent. The absence of a noticeable increase in distractor interference when VWM-matching is highly surprising, as it is at odds with a substantial body of work showing automatic guidance by irrelevant features stored in VWM (for reviews see Soto et al., 2008; Oberauer, 2019). There are, however, several key differences in the design from this previous research, meaning that VWM-driven capture by affective categories could be contingent upon other factors.

## Experiment 2

Another stimulus-related explanation for the lack of VWM driven capture in Experiment 1 could be that the capture may be dependent upon the degree the task enabled processing of the VWM-matching distractor dimension. Indeed, earlier studies often presented the irrelevant VWM-matching stimulus for a longer duration (e.g., Bahle et al., 2020; Olivers et al., 2009 ), allowing longer processing of the irrelevant stimulus’s features. The 100ms presentation in a peripheral task-irrelevant location may therefore have reduced the salience of the affective/conceptual content to interact with the representation in VWM, despite its affective associations. To test whether VWM-driven effects emerge for more when the VWM-matching distractors’ content is more visible and salient, the distractor remained on the screen beyond the presentation of the letter array to increase its salience relative to the target stimuli.

### Methods

#### Participants

An initial sample of 29 participants was recruited, though after application of the pre-registered exclusion criteria, 4 were excluded due to poor performance on either the visual search or VWM change detection task (scoring < 0.60 accuracy ) or failing to complete the experiment due to technical error. The minimum pre-registered sample was 24 participants (see pre-registration: https://osf.io/dyb3u) based on the same sample size selection rationale outlined in Experiment 1, though to fulfil all confirmed participant bookings, an additional participant was tested. The final sample of 25 participants consisted of 14 women and 11 men. The average age of participants was 23.84 years, *SD* = 4.62. Twenty of the 25 participants were right-handed. The average level of trait anxiety was 46.80, *SD* = 9.64, and state anxiety was 36.72, *SD* = 9.81. Average spider fear score was 2.76, *SD* = 1.93.

#### Stimuli and procedure

The task was nearly identical to Experiment 1, with the exception that the distractor remained on screen after the 100ms letter array had been presented for the full duration of the response window or until a response was made.

The computer display was also changed for Experiment 2 (as well as all subsequent Experiments), and stimuli were now presented on a 21.5-inch HP EliteDisplay E221c display with a 60 Hz refresh rate and 1920 × 1080 resolution.

## Results

**Fig. 4:**
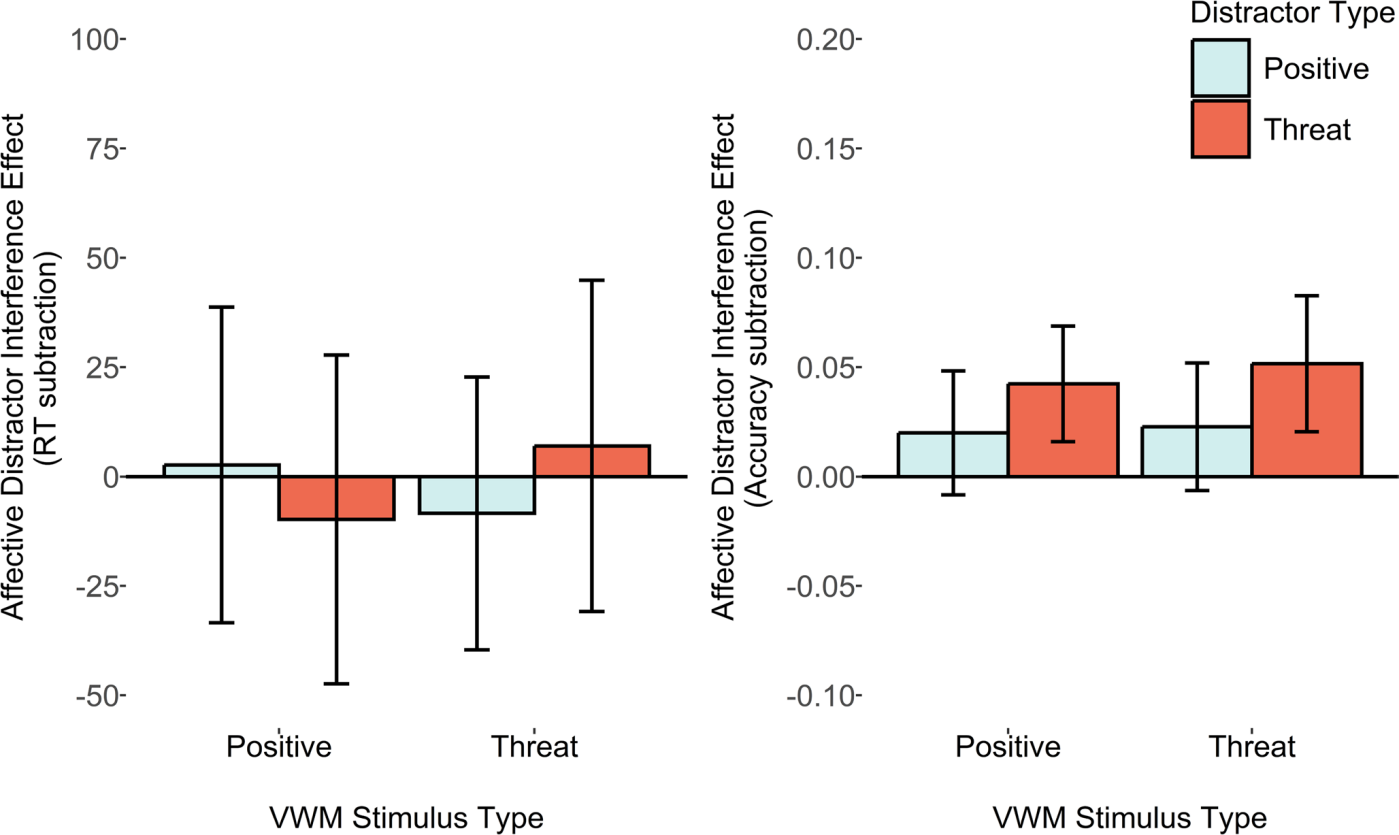
Plots depicting average reaction time and accuracy affective distractor interference scores (relative to a neutral distractor condition). The legend depicts different peripheral distractor types, and error bars represent within-subjects 95% confidence intervals

The key pre-registered pairwise comparisons again were the comparison of the ADI_RT_ scores between VWM-match and mismatch conditions (see Fig. 4). For the threat-related ADI_RT_ score, the Bayes factors computed with the reaction time based measure showed inconclusive evidence favouring neither hypothesis strongly, *M*_*diff*_ = 17ms, *SD*_*diff*_ = 152, *d*_*z*_ = 0.11, B_H[0, 28]_ = 1.02, but did show substantial evidence (i.e., BF < 0.33) favouring the null hypothesis for the accuracy based ADI_accuracy_ measure, *M*_*diff*_ = − 0.003, *SD*_*diff*_ = 0.11, *d*_*z*_ = − 0.03, B_U[0, 0.12]_ = 0.21.

Similarly, for the positive distractor ADI_RT_ score the Bayes factors computed with the reaction time based measure showed inconclusive evidence favouring neither the null or experimental hypothesis strongly, *M*_*diff*_ = 11ms, *SD*_*diff*_ = 134, *d*_*z*_ = 0.08, B_H[0, 28]_ = 0.89, but did show substantial evidence favouring the null with the ADI_accuracy_ score comparison, *M*_*diff*_ = 0.01, *SD*_*diff*_ = 0.11, *d*_*z*_ = 0.08, B_U[0, 0.12]_ = 0.33.

The follow-up comparisons for each ADI_RT_ score (see Table 3), showed a lack of substantial evidence of interference from either positive or threat-related distractors, relative to neutral distractors, across all conditions, *d*_z_’s < 0.11, B_H[0, 28]_ = 0.38 − 0.77. Analysis of the ADI_accuracy_ score, however, revealed substantial evidence of increased errors when the spider threat-related distractor was presented, versus the neutral bird, within both VWM-match, *d*_*z*_ = 0.48, B_U[0, 0.12]_ = 8.22, and mismatch conditions, *d*_*z*_ = 0.60, B_U[0, 0.12]_ = 28.21. There was, however, inconclusive evidence of increased interference from the positive distractors, *d*_*z*_’s < 0.34, B_U[0, 0.12]_ = 0.69 − 0.97.

**Table 3:** Means and standard deviations for reaction time (RT) and accuracy (probability), and affective distractor interference (ADI) scores, as well as accuracy in the change detection task. Bayes factors are reported with priors listed in notation. Bayes factors showing substantial evidence for the experimental hypothesis (i.e., BF > 3) are highlighted in bold

	VWM	Distractor	RT (SD)	ADI_RT_ score	B_H[0, 28]_	Accuracy (SD)	ADI_accuracy_ score	B_U[0, 0.12]_	VWM (SD)
Experiment 2 (*n* = 25)	Positive	Neutral	929 (217)	-	-	0.91 (0.09)	-	-	0.78 (0.11)
		Positive	932 (208)	3 (79)	0.56	0.88 (0.09)	0.02 (0.06)	0.97	0.77 (0.08)
		Threat	919 (190)	−10 (88)	0.38	0.87 (0.08)	**0.04 (0.07)**	**28.21**	0.78 (0.08)
	Threat	Neutral	898 (167)	-	-	0.89 (0.07)	-	-	0.77 (0.10)
		Positive	890 (185)	−8 (100)	0.45	0.87 (0.10)	0.02 (0.11)	0.69	0.76 (0.09)
		Threat	905 (199)	7 (114)	0.77	0.84 (0.11)	**0.05 (0.11)**	**8.22**	0.71 (0.10)

### VWM performance

As 2 × 3 repeated measures ANOVA conducted on the VWM change detection performance revealed that there was a significant difference between the VWM types, *F*(1,24) = 7.45, *p* =.012, *ƞ*^*2*^_*p*_ = 0.24, with the change to the spider VWM cues less accurately detected. The difference across distractors was non-significant, *F*(2,48) = 2.50, *p* =.093, *ƞ*^*2*^_*p*_ = 0.09, though the interaction was significant, *F*(2,48) = 4.25, *p* =.020, *ƞ*^*2*^_*p*_ = 0.15. Comparing the difference in accuracy for each of the distractors across the two VWM conditions revealed that the spider distractor resulted in lower VWM change detection performance in the threat-related spider condition, *t*(24) = 4.28, *p* <.001, *d*_*z*_ = 0.87. This is likely due to the visual similarity of the spider image selectively disrupting the representation of the different spider exemplar in VWM, versus the visually distinct kitten exemplar (Zhang & Peacock, 2025).

Conversely, there was no difference in change detection performance between positive and threat-related categories when the distractor was a bird and kitten, *p* >.408.

## Discussion

The evidence of attentional capture in the ADI_accuracy_ score is consistent with evidence showing that task-irrelevant spider stimuli disrupt visual search and capture attention (Gerdes et al., 2008; Mogg & Bradley, 2006). Further, the VWM change detection data suggests that the identity of the distractors was also processed to later stages, as indicated by the reduce performance on the spider change detection block. It suggests that the current task is sensitive to value-driven attentional capture. This same effect, however, was not found for the positive kitten distractors, despite them being rated as more positive and emotionally arousing than the neutral distractors (see Table 2).

Against the main experimental hypothesis there was no evidence of VWM-driven attentional capture. One possible reason for this could be that participants were able to effectively suppress the distractor locations, as evidence has shown that participants are able to prevent VWM guidance when the distractor appears in a peripheral task-irrelevant location (Hernandez et al., 2010; Pan & Soto, 2010; Yang et al., 2024). Indeed, most studies showing guidance of attention by VWM representations present the distractor in a potential target location (e.g., Bahle et al., 2020). The evidence of increased processing of the spider distractors from visual search accuracy and VWM change detection accuracy, however, indicates that the absence of VWM-guidance is unlikely to be because the distractor locations were effectively inhibited – contradicting spatial suppression as a potential explanation for the null result. The current findings, therefore, suggest another factor limiting the memory-driven capture, or could point to potential confounds in the experimental design.

## Experiment 3

To effectively explore the cognitive mechanisms by which VWM representations drive involuntary attentional capture (or not in this case), possible task confounds must first be discounted. One possible reason for this lack of VWM-driven distractor interference could be due to the task-demands of the VWM change detection task itself. Evidence has shown that guidance from top-down representations requires sufficient detail to be active as an attentional set, and for this to overlap with the distractor features (Kerzal & Witzel, 2019; Williams et al., 2022); however, participants often do not represent target objects with exact fidelity in VWM, especially if they’re not prioritised, and instead maintain them in their simplest form that still enables sufficient overall task performance (Bays et al., 2009; Zhang & Luck, 2008; for review Yu et al., 2023). It is possible that participants may not have represented the specific category defining features of the animals in VWM, and instead merely maintained them as an imprecise shape, as this was sufficient to perform the change detection task. This lack of specificity could then cause capture by all distractors equally, obscuring category specific VWM-driven capture.

To rule out this possibility, additional baseline conditions were included in Experiment 3. These included a condition without the VWM change detection task, where participants completed the visual search task in isolation; as well as a no distractor condition, in which no peripheral distractor was presented in addition to neutral and threat-related distractors.

These new baseline conditions allowed pre-registered specialised pairwise comparisons to rule out potential confounds (see pre-registration: https://osf.io/98kz7). To enable these comparisons, distractor interference (DI) scores, rather than ADI scores, were computed, which contrasted the distractor present conditions to the no distractor conditions. Higher value DI scores are indicative of greater interference from a distractor in visual search, regardless of its affective or conceptual content.

The first set of pre-registered comparisons was designed to test for VWM-content specific capture, specifically testing whether retaining a particular animal in VWM resulted in increased capture by other exemplars of that animal. This was similar to the previous comparisons of ADI score between VWM-matching versus VWM-mismatching conditions, but using the no distractor baseline condition rather than the neutral distractor baseline condition to compute a DI score. If there was content-specific capture for the spider distractors, then it would be expected that the DI score for the spider (i.e., spider versus no distractor reaction time) would be higher when the VWM change detection stimuli were spiders (i.e., matching) versus when they were kittens (i.e., mismatching); and that a similar increase in distractor interference between these conditions would not be observed for the DI score computed for the bird stimuli.

The next set of pre-registered comparisons was to test the general influence of the VWM task on overall distractor interference. If the null effect observed were due to the general influence of maintaining a stimulus in VWM then it would be expected that performance of the VWM change detection task with both positive and threat-related animals would result in a larger DI score relative to the no VWM task baseline, regardless of the type of animal distractor.

To further test if all distractor interference was caused by maintaining any stimulus in VWM during the visual search, both neutral and threat-related distractors were compared to the no distractor baseline in the no VWM task condition in isolation. If even ostensibly goal-independent attentional capture by the distractors was actually caused by maintaining any stimulus in VWM, then it would be expected that when the change detection task was removed that there would be no evidence of distractor interference.

### Methods

#### Participants

Based on the same sample size selection rationale outlined in Experiment 1, a sample of 24 participants was pre-registered. An initial sample of 28 participants was recruited, though after application of the pre-registered exclusion criteria (see pre-registration: https://osf.io/98kz7), 4 were excluded due to poor performance on either the search or VWM change detection task (scoring < 0.60 accuracy) or failing to complete the experiment due to technical error. The final pre-registered sample of 24 consisted of 19 women and 5 men. The average age of participants was 22.42 years, *SD* = 7.13. Twenty-one of the 24 participants were right-handed. The average level of trait anxiety was 44.17, *SD* = 9.68, and state anxiety was 37.25, *SD* = 9.26. Average spider fear score was 2.87, *SD* = 1.38.

#### Stimuli and procedure

The task and procedure in Experiment 3 was similar to Experiment 1, though to create a suitable baseline condition to control for potential confounds, the positive kitten distractor condition was replaced by a distractor absent condition where no peripheral distractors were presented. As in Experiment 1, the letter array and distractors were presented for 100ms.

Additionally, a no VWM change detection task condition was also included alongside the kitten and spider VWM change detection conditions. Within these blocks of trials, participants first viewed the fixation dot on screen for 900ms, before the 100ms visual search display appeared, followed by the 2500ms blank screen response window. After the response window duration, or until a response was made, a 100ms blank screen with auditory error feedback was then presented, followed by a 200ms blank ISI, and 200ms white noise end-of-trial cue.

The task was therefore composed of six 72-trial blocks, resulting in 48 trials for each of the nine conditions in the 3 × 3 repeated measures design. All conditions were counterbalanced within each block, apart from block order which was counterbalanced between participants. Within this version of the task, the practice block was extended to include an additional block of 12 trials with only the visual search task, with no concurrent VWM change detection task.

## Results

### Testing for VWM content-specific effects on distractor interference

Comparing the effect of VWM congruence on the threat-related DI score (i.e., distractor present versus distractor absent) by contrasting the spider DI score between threat VWM-matching and positive VWM-mismatching conditions revealed inconclusive evidence of a difference for the DI_RT_ score, *M*_*diff*_ = 14ms, *SD*_*diff*_ = 111, *d*_*z*_ = 0.13, B_H[0, 28]_ = 0.98, and anecdotal evidence favouring the null for the DI_accuracy_ score, *M*_*diff*_ = 0.01, *SD*_*diff*_ = 0.06, *d*_*z*_ =0.23, B_U[0, 0.12]_ = 0.39.

As a comparison, repeating this contrast between the positive kitten VWM and threat-related spider VWM conditions but with the neutral bird DI score revealed a similar pattern, with no substantial evidence of a difference for DI_RT_ score, *M*_*diff*_ = 35ms, *SD*_*diff*_ = 118, *d*_*z*_ = 0.30, B_H[0, 28]_ = 2.11, though there was strong evidence against a decrease in the DI_accuracy_ score, *M*_*diff*_ = − 0.003, *SD*_*diff*_ = 0.05, *d*_*z*_ = -0.07, B_U[0, 0.12]_ = 0.09.

The comparison between these two effects showed substantial evidence that the threat-related spider VWM task condition failed to increase distractor interference for the spider distractor any more than the bird distractor, *M*_*diff*_ = −21ms, *SD*_*diff*_ = 82, *d*_*z*_ = − 0.26, B_H[0, 28]_ = 0.26. Indeed, purely numerically, the neutral bird distractor actually showed a larger increase in DI_RT_ score; though a follow-up exploratory two-tailed pairwise comparison between these effects to test for differences in the opposing direction to the hypothesis revealed no significant difference, *t*(23) = 1.25, *p* =.223. The comparison with DI_accuracy_ score as the outcome measure revealed only anecdotal evidence favouring the experimental hypothesis, *M*_*diff*_ = 0.02, *SD*_*diff*_ = 0.06, *d*_*z*_ = 0.33, B_U[0, 0.12]_ = 0.85.

### Testing for general VWM effects on distractor interference

Pre-registered pairwise comparisons were also conducted to assess evidence for the general influence of VWM contents on processing of peripheral distractors. Average spider DI scores across both kitten and spider VWM conditions, versus spider DI_RT_ scores in the no VWM task condition, revealed that there was no evidence that the VWM task increased distractor interference on visual search reaction time, *M*_*diff*_ = 4ms, *SD*_*diff*_ = 50, *d*_*z*_ = 0.08, B_H[0, 28]_ = 0.46, and strong evidence that it had no effect on the DI_accuracy_ scores, *M*_*diff*_ = − 0.003, *SD*_*diff*_ = 0.05, *d*_*z*_ = − 0.07, B_U[0, 0.12]_ = 0.08.

**Fig. 5:**
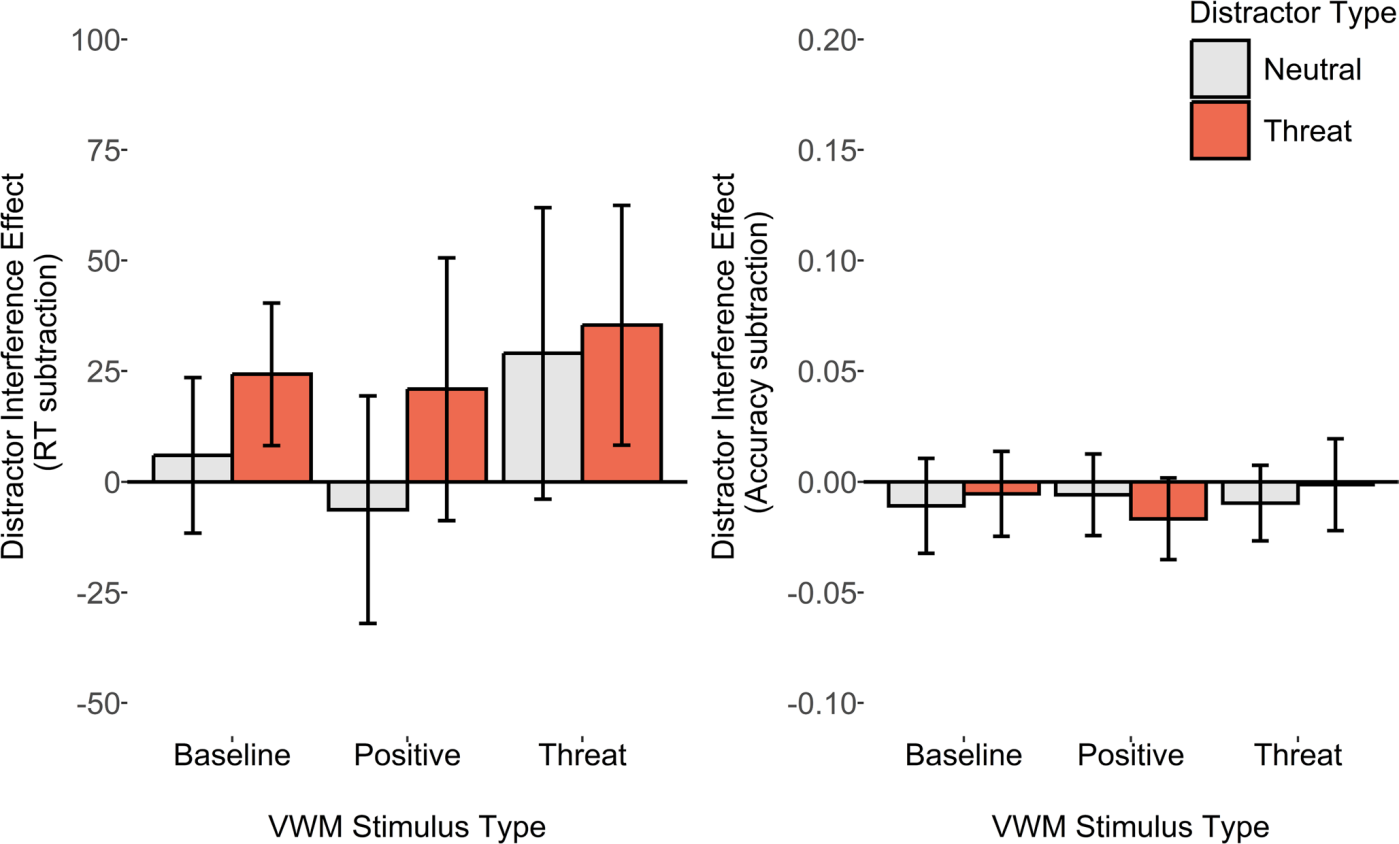
Plots depicting average reaction time and accuracy distractor interference scores (relative to a no distractor condition). Error bars represent within-subjects 95% confidence intervals

Similarly, there was no substantial evidence that the VWM task increased interference from neutral distractors, for DI_RT,_ *M*_*diff*_ = 5ms, *SD*_*diff*_ = 52, *d*_*z*_ = 0.10, B_H[0, 28]_ = 0.54, and moderate evidence that it had no effect on DI_accuracy_, *M*_*diff*_ = 0.003, *SD*_*diff*_ = 0.06, *d*_*z*_ = 0.05, B_U[0, 0.12]_ = 0.16. Thus, it appears that the VWM load did not strongly increase or decrease the susceptibility to distraction in the current task (Fig. 5).

### Testing for purely VWM-independent affective attentional capture

To further test whether the VWM change detection task influenced attentional capture by the animal distractors, the reaction time when the distractor was neutral and when it was threat-related were both compared to the baseline no distractor condition on blocks without the VWM task. The results are displayed in Table 4, and reveal that there was substantial evidence that the threat-related spider distractor disrupted visual search, conversely, there was no evidence suggesting attentional capture by the neutral bird distractor with anecdotal evidence favouring the null hypothesis. As can be seen from Table 4, the same comparisons with accuracy data were near zero and favoured the null hypothesis.

**Table 4:** Means and standard deviations for reaction time (RT) and accuracy (probability), and distractor interference (DI) scores, as well as accuracy in the change detection task. Bayes factors are reported with priors listed in notation. Bayes factors showing substantial evidence for the experimental hypothesis (i.e., BF > 3) are highlighted in bold

	VWM	Distractor	RT (SD)	DI_RT_ score	B_H[0, 28]_	Accuracy (SD)	DI_accuracy_ score	B_U[0, 0.12]_	VWM (SD)
Experiment 3 (*n* = 24)	No VWM	No distractor	660 (112)	-	-	0.91 (0.06)	-	-	-
		Neutral	666 (111)	6 (41)	0.54	0.90 (0.08)	0.01 (0.05)	0.32	-
		Threat	685 (127)	**24 (48)**	**9.94**	0.91 (0.06)	0.01 (0.04)	0.16	-
	Positive	No distractor	863 (192)	-	-	0.92 (0.08)	-	-	0.76 (0.07)
		Neutral	856 (172)	−6 (61)	0.29	0.91 (0.08)	0.01 (0.05)	0.18	0.81 (0.08)
		Threat	884 (193)	21 (82)	1.57	0.90 (0.08)	0.02 (0.06)	0.62	0.80 (0.07)
	Threat	No distractor	829 (146)	-		0.90 (0.08)	-	-	0.83 (0.09)
		Neutral	858 (172)	29 (84)	2.70	0.89 (0.09)	0.01 (0.04)	0.28	0.78 (0.10)
		Threat	864 (168)	**35 (67)**	**12.63**	0.90 (0.08)	< 0.01 (0.05)	0.13	0.80 (0.11)

A follow-up unregistered analysis exploring the goal-independent ADI effects was conducted comparing the magnitude of the neutral DI score and threat-related DI score in the no VWM condition. This revealed moderate evidence that the threat-related distractor cause more slowing relative to the neutral distractor, *M*_*diff*_ = 18ms, *SD*_*diff*_ = 44, *d*_*z*_ = 0.42, B_H[0, 28]_ = 4.01. Repeating this exploratory analysis with all neutral versus threat-related DI scores collapsed across VWM conditions replicated this general value-driven ADI effect, *M*_*diff*_ = 17ms, *SD*_*diff*_ = 35, *d*_*z*_ = 0.50, B_H[0, 28]_ = 7.92.

### VWM performance

Assessing the accuracy of the VWM change detection accuracy across the three distractor types in the kitten and spider VWM conditions revealed no difference in VWM type, *F*(1,23) = 1.38, *p* =.252, *ƞ*^*2*^_*p*_ = 0.06, or distractor type, *F*(2,46) = 0.22, *p* =.800, *ƞ*^*2*^_*p*_ = 0.01. There was however a significant interaction between VWM and distractor type, *F*(2,46) = 0.9.36, *p* <.001, *ƞ*^*2*^_*p*_ = 0.29. Comparison of the change detection accuracy between VWM types for each distractor revealed that the difference was specific to the baseline no distractor condition, with accuracy being lower for the kitten VWM stimuli, *t*(23) = 4.34, *p* <.001, *d*_*z*_ = 0.89, and that there was no significant difference between VWM types when the distractor was a bird or spider, *p* >.136.

## Discussion

Across three experiments there was no evidence of the hypothesised VWM-driven attentional capture by the task-irrelevant affective distractors. Whilst Bayes factors didn’t strongly favour the null hypothesis, the consistent inconclusive evidence is at odds with the extensive literature showing evidence of VWM-driven capture, which the samples were powered to detect.

Experiment 3 rules out several interpretations for the recorded null effects. Specifically, the replicated null finding comparing VWM-matching to mismatching conditions but with a no distractor baseline, indicates that the effect wasn’t due to the selection of the neutral bird stimuli as the comparison distractor. Further, this experiment also demonstrates that the lack of VWM-driven capture was not due to a non-specific template causing increased interference by all distractors, as there was no substantial evidence of a difference from a condition without the VWM task.

Examination of the performance on the change detection task also shows that performance across all experiments for both VWM animal types is well above chance (i.e., accuracy > 0.75), indicating that participants were engaging with the task and retaining the specific stimulus features in VWM on most trials. It should be noted, however, that accuracy on the change detection task does not necessarily indicate that these remembered stimuli were stored in a prioritised state across the whole trial. Evidence has suggested that involuntary capture of attention by VWM-matching stimuli is contingent upon the active prioritization of this stimulus, relative to other competing targets (Olivers et al., 2011). For instance, it has been found that VWM-matching distractors often only capture attention when presented in isolation, where they do not compete with more relevant target stimuli (Downing & Dodds, 2004; Houtkamp & Roelfsema, 2006). Additionally, models of working memory have posited that a stimulus can be retained in VWM in a de-prioritised state when not immediately relevant, where it may not influence the selection of visually congruent stimuli (Hollingworth & Hwang, 2013), or visual search speed, generally (Hollingworth & Maxcey-Richard, 2013). See General Discussion for more information on this point.

The design of the experiments may therefore have prevented the representation in VWM guiding attention. Specifically, the distractor was always presented concurrent to the target stimulus meaning that the more relevant target letter representation could outcompete the guidance from the contents of VWM. Further, the consistent structure of the trial would likely facilitate this, as participants would be aware that they would perform the tasks in sequence, reducing the need to prioritise the features in VWM during the onset of the task-irrelevant distractors.

## Experiment 4

In order to test whether the VWM-driven capture was dependent upon prioritisation of the affective features in VWM, the temporal structure of the trial was altered to limit participants’ ability to perform the tasks in sequence. To do this, the VWM probe and the visual search target were presented in a random order with an equal probability of appearing after the VWM cue, meaning that both tasks were equally relevant and thus should be prioritised equally in attention.

### Methods

#### Participants

Based on the same sample size selection rationale outlined in Experiment 1, a sample of 24 participants was pre-registered. An initial sample of 27 participants was recruited, though after application of the pre-registered exclusion criteria, 3 were excluded due to poor performance on either the search or VWM change detection task (scoring < 0.60 accuracy) or failing to complete the experiment due to technical error. The final pre-registered sample of 24 participants consisted of 20 women and 4 men. The average age of participants was 21.67 years, SD = 4.90. Seventeen participants of the 24 were right-handed. The average level of trait anxiety was 42.08, SD = 12.13, and state anxiety was 36.04, *SD* = 9.69. The average spider fear score was 2.63, SD = 1.47.

#### Stimuli and materials

Within Experiment 4, the main difference was changing the fixed sequential order of the concurrent VWM change detection task and visual search task. Each block now consisted of 144 trials, 72 of which were change detection trials, and 72 were visual search trials.

For both trial types, the trial began with a 900ms fixation dot, followed by a VWM cue for 500ms, then another 500ms fixation dot. For the visual search task trials, specifically, the letter array and peripheral distractor appeared for 100ms, followed by a 2500ms response window. After the response window duration, or a response was made, a 100ms blank screen was presented with brief auditory feedback on error trials, followed by the 200ms white noise end-of-trial cue.

For the VWM change detection trials, after the second fixation dot, the VWM probe image was presented in a central position. To ensure that VWM probe image was clearly distinct from the distractor image, which now had an equal probability of appearing after the VWM cue, it was reduced in size to 2.30° × 2.30° square in the central position. This was in the identical area of the letter array, further distinguishing the task-relevant area. Additionally, a black rectangle the same size as the peripheral distractor was presented in the same possible locations to the distractors (50% of trials to the left, 50% to the right). The black rectangle was designed to highlight the spatially irrelevant location across both forms of trials, and to clearly discriminate the VWM trials from the visual search trials. The VWM probe image appeared for 100ms on the screen, after which participants had 2500ms to respond. This was followed by the 500ms text feedback to their response, and the 200ms white noise end-of-trial cue.

These trials were presented in a random order within each block, thus requiring participants to prioritise both tasks equally. As before, in two of the four blocks the VWM cue/probe was a kitten, and the other two a spider. These were presented in an alternating order, with the order counterbalanced between participants.

To ensure that participants could prioritise responding to the VWM change detection task as well as the visual search, they were instructed to respond to the visual search trials with the righthand keypad (rather than keyboard number line) using the ‘0’ and ‘2’ keys, and respond to the change detection task with their left hand using the S and D keys. They were encouraged to respond as quickly as possible to both tasks. All other measures were identical to the earlier Experiments.

## Results

**Fig. 6:**
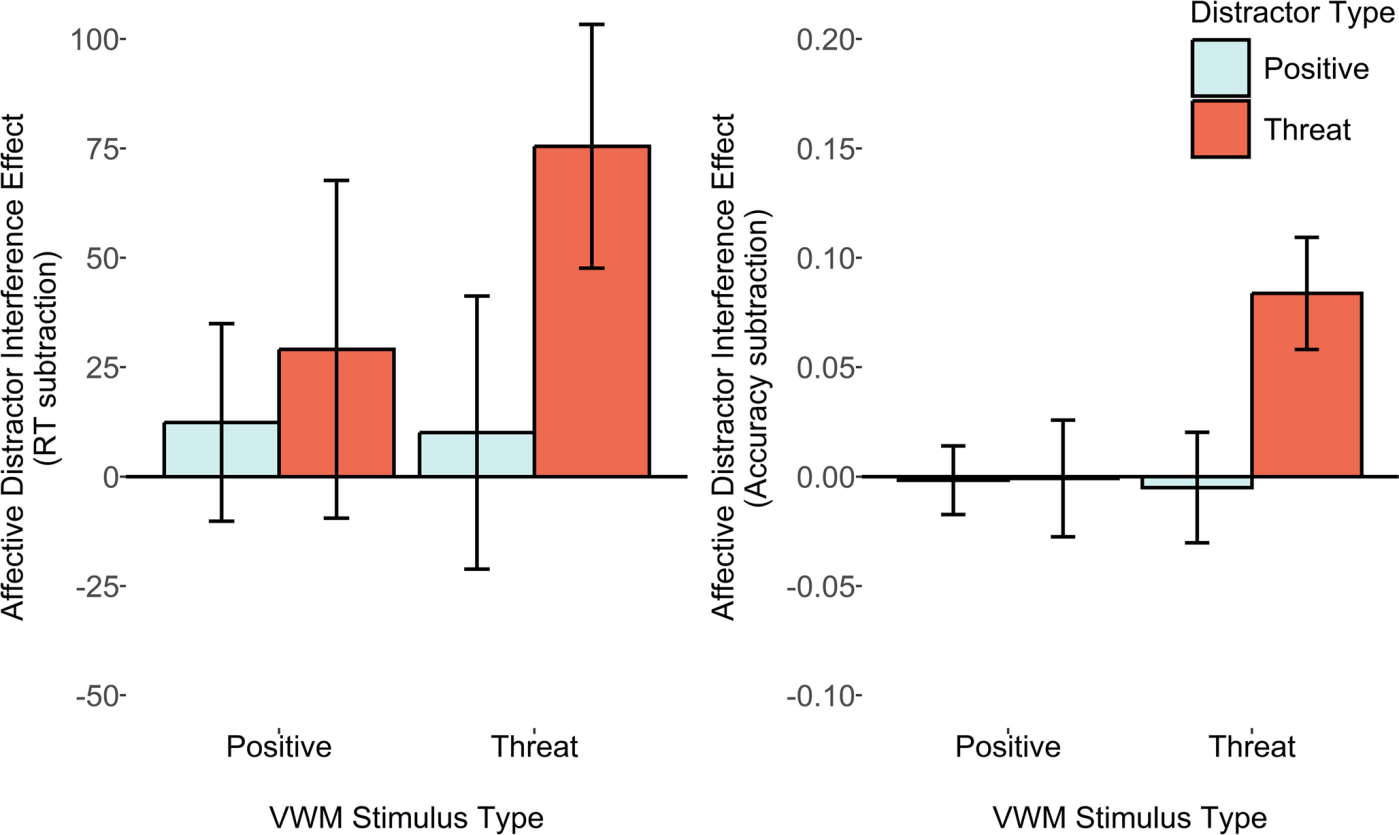
Plots depicting average reaction time and accuracy affective distractor interference scores (relative to a neutral distractor condition). Error bars represent within-subjects 95% confidence intervals

The key pre-registered ADI score comparisons revealed that, in contrast to earlier results, there was now moderate evidence that the VWM-matching threat-related distractors resulted in a larger ADI_RT_ score relative to the VWM-mismatching condition, with reaction time data, *M*_*diff*_ = 46ms, *SD*_*diff*_ = 126, *d*_*z*_ = 0.37, B_H[0, 28]_ = 3, and extreme evidence with accuracy-based ADI_accuracy_ scores, *M*_*diff*_ = 0.08, *SD*_*diff*_ = 0.10, *d*_*z*_ = 0.88, B_H[0, 28]_ = 4164.55. For the positive distractors, however, there was only anecdotal evidence favouring the null, for the reaction time ADI_RT_ score, *M*_*diff*_ = 2ms, *SD*_*diff*_ = 86, *d*_*z*_ = 0.02, B_H[0, 28]_ = 0.19, and moderate evidence favouring the null for accuracy-based ADI_accuracy_ scores, *M*_*diff*_ < 0.01, *SD*_*diff*_ = 0.07, *d*_*z*_ = 0.05, B_U[0, 0.12]_ = 0.19 (Fig. 6).

Assessment of the individual reaction time ADI_RT_ scores within both VWM-matching and mismatching conditions (see Table 5), revealed only weak anecdotal evidence of a difference between affective and neutral distractors. The exception to this was the threat-related distractor in the VWM-matching condition, which showed extremely strong evidence of slower target detection for the threat-related distractor.

**Table 5:** Means and standard deviations for reaction time (RT) and accuracy (probability), and affective distractor interference (ADI) scores. Bayes factors are reported with priors listed in notation. Bayes factors showing substantial evidence for the experimental hypothesis (i.e., BF > 3) are highlighted in bold

	VWM	Distractor	RT (SD)	ADI_RT_ score	B_H[0, 28]_	Accuracy (SD)	ADI_accuracy_ score	B_U[0, 0.12]_
Experiment 4 (*n* = 24)	Positive	Neutral	932 (163)	-	-	0.87 (0.10)	-	-
		Positive	944 (161)	12 (51)	1.12	0.88 (0.10)	0 (0.07)	0.13
		Threat	961 (185)	29 (82)	2.89	0.87 (0.09)	0 (0.08)	0.15
	Threat	Neutral	934 (159)	-	-	0.89 (0.07)	-	-
		Positive	944 (200)	10 (79)	0.82	0.90 (0.08)	− 0.01 (0.05)	0.08
		Threat	1009 (192)	**75 (70)**	**65305.44**	0.81 (0.12)	.**08 (0.07)**	**25700984.91**

A similar pattern was found for the accuracy-based ADI_accuracy_ score, with the VWM-matching threat-related distractor condition being the only one showing evidence of poorer accuracy versus the neutral distractor. All other conditions showed substantial evidence favouring the null.

### VWM performance

Comparison of the accuracy on the VWM change detection trials between animal types revealed that performance was superior with positive kitten stimuli, *M* = 0.90, *SD* = 0.06 relative to the threat-related spider stimuli, *M* = 0.85, *SD* = 0.09, *t*(23) = 3.26, *p* =.003, *d*_*z*_ = 0.67.

## Discussion

The results show that requiring participants to prioritise both change detection and visual search tasks equally resulted in the hypothesised VWM-driven capture from threat-related distractors. Such a result is consistent with models which propose that stimuli can be flexibly maintained in different states within VWM, only guiding attention when they are actively prioritised due to task demands (Hollingworth & Hwang, 2013; Kerzel & Witzel, 2019; van Moorselaar et al., 2014; 2015; see General Discussion for further detail).

Experiment 4 did, however, also differ in the participants’ ability to prioritise the response to the change detection task, relative to previous experiments: Participants were encouraged to prepare speeded responses to both tasks, and the response button placement allowed action preparation to both tasks with separate hands. Conversely, In Experiments 1–3 the button placement for earlier experiments prevented participants from preparing a response to the VWM task, as the spacing of the keys required participants to use both hands to perform the visual search response, before switching to the change detection response. Evidence has shown that the action planning with a specific motor response disrupts the preparation of future action plans also requiring that motor response (Stoet et al., 1999). Furthermore, participants in Experiment 4 were encouraged to respond quickly to the change detection task, whilst in Experiments 1–3 there were instructed to prioritise accuracy reducing the need to prepare an action.

Research has long shown a link between action and attention, whereby manipulations of response type and preparation modulate the priority of action relevant items in attention and working memory (Allport, 1987; Hommel, 2004; Tipper et al., 1998). More recent evidence has shown that action planning and preparation may influence attention by acting on the preparatory state of the stimulus representation in VWM (for reviews see Heuer et al., 2020; Olivers & Roelfsema, 2020; van Ede, 2020). For example, van Ede et al. (2019) found that when participants retained multiple stimuli in VWM requiring different responses, neural activity coupled with each stimulus’ location was equal if they were unable to initiate the response; however, when a stimulus was cued as the target and participants could respond towards it, neural activity increased specifically for the target stimulus’ location – consistent with action preparation facilitating specific stimulus prioritisation in VWM. Further, studies have also shown that preparation of motor actions towards a specific stimulus increases processing of that stimulus, even when it is currently task-irrelevant (Trentin et al., 2023).

## Experiment 5

Experiment 5 was therefore designed to test whether increased capture by the VWM-matching distractor was due entirely to the altered trial structure changing stimulus onset expectations, or whether action preparation played a role. This was a near identical replication of Experiment 1, except that participants were now encouraged to respond as fast as possible to both tasks, and the response button layout was altered to allow action preparation for both tasks with different hands. Thus, the fixed temporal structure of the trial remained intact, whilst the ability and motivation to prepare an action to the affective stimuli in VWM was increased.

### Methods

####  Participants

Based on the same sample size selection rationale outlined in Experiment 1, a sample of 24 participants was pre-registered. An initial sample of 26 participants was recruited, though after application of the pre-registered exclusion criteria 2 were excluded due to poor performance on either the search or VWM change detection task (scoring < 0.60 accuracy; see pre-registration: http://osf.io/pzb26). The final pre-registered sample of 24 participants consisted of 22 women and 2 men. The average age of participants was 21.25 years, *SD* = 3.10. Twenty-three participants of the 24 participants were right-handed. The average level of trait anxiety was 42.88, *SD* = 10.40, and state anxiety was 37, *SD* = 10.68. The average spider fear score was 3.24, *SD* = 1.88.

#### Stimuli and procedure

The task was nearly identical to Experiment 1, the exceptions being that participants were encouraged to make a speeded response to both the visual search task and the VWM change detection task. To enable action preparation of the VWM task, participants responded to the visual search task with the ‘0’ and ‘2’ keys on the right keypad, and the change detection task with the ‘s’ and ‘d’ keys using their left hand, as in Experiment 4.

## Results

The pre-registered comparison of the reaction time ADI_RT_ scores revealed moderate evidence favouring the experimental hypothesis, showing that VWM-matching threat-related distractors caused more interference, versus when VWM-mismatching, *M*_*diff*_ = 48ms, *SD*_*diff*_ = 99, *d*_*z*_ = 0.49, B_H[0, 28]_ = 7.44. For the secondary ADI_accuracy_ score comparison, there was only anecdotal evidence favouring the null hypothesis, *M*_*diff*_ = − 0.01, *SD*_*diff*_ = 0.06, *d*_*z*_ = − 0.17, B_U[0, 0.12]_ = 0.08.

For the comparison of positive ADI_RT_ score, between VWM-matching and mismatching conditions, there was only anecdotal evidence favouring the null, for both the reaction time based score, *M*_*diff*_ = −13ms, *SD*_*diff*_ = 94, *d*_*z*_ = − 0.14, B_H[0, 28]_ = 0.38, and ADI_accuracy_ score, *M*_*diff*_ = 0.02, *SD*_*diff*_ = 0.08, *d*_*z*_ = 0.21, B_U[0, 0.12]_ = 0.49.

**Fig. 7:**
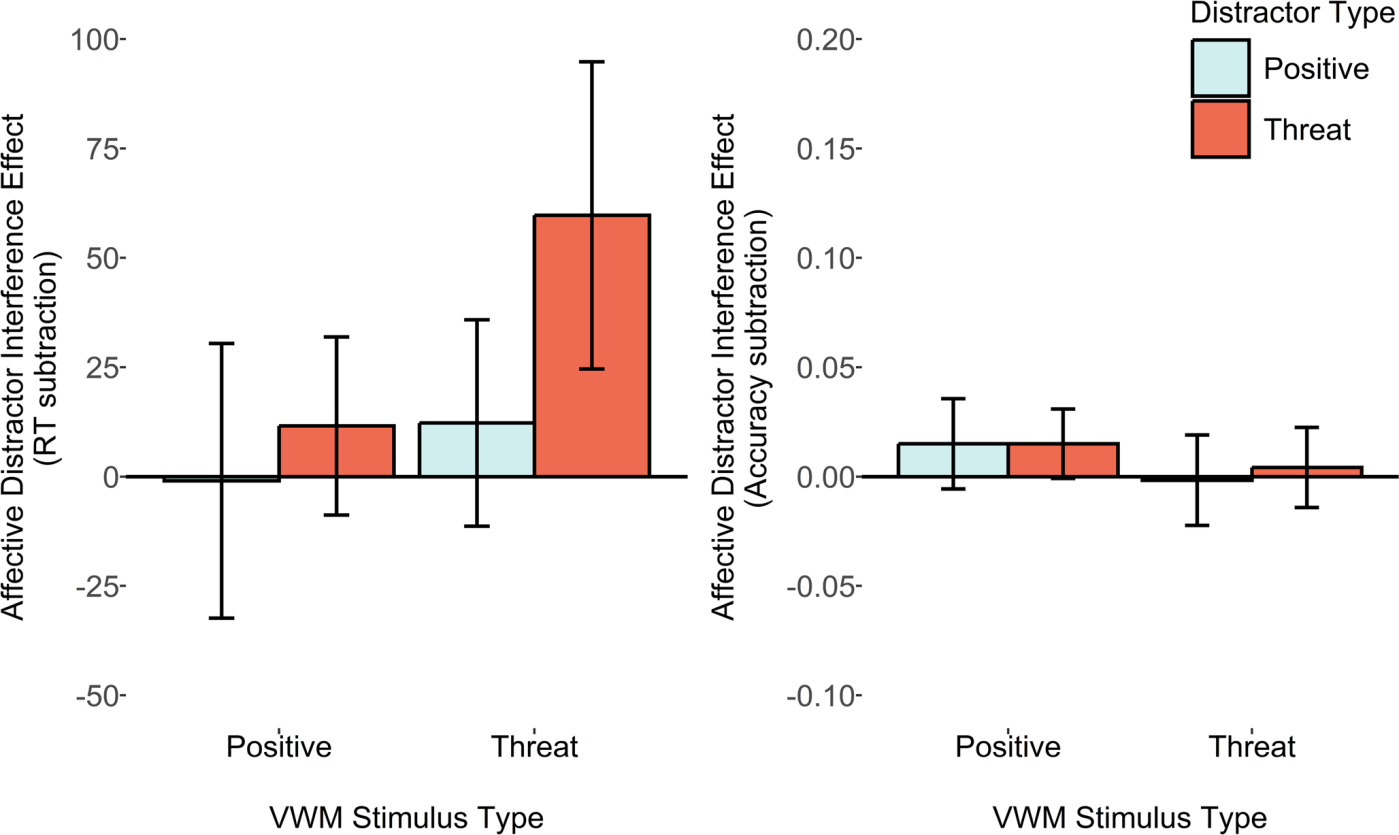
Plots depicting average reaction time and accuracy affective distractor interference scores (relative to a neutral distractor condition). Error bars represent within-subjects 95% confidence intervals

Assessment of the individual ADI scores within both VWM-matching and mismatching conditions separately (see Table 6), revealed anecdotal evidence of a difference between affective and neutral distractors across most conditions. The exception being within the VWM-matching threat condition, which showed strong evidence of affective interference. For the accuracy-based ADI scores, all Bayes factors favoured the null hypothesis, with the ADI scores in the spider VWM condition showing strong evidence for the null (Fig. 7).

**Table 6:** Means and standard deviations for reaction time (RT) and accuracy (probability), and affective distractor interference (ADI) scores, as well as accuracy in the change detection task. Bayes factors are reported with priors listed in notation. Bayes factors showing substantial evidence for the experimental hypothesis (i.e., BF > 3) are highlighted in bold

	VWM	Distractor	RT (SD)	ADI_RT_ score	B_H[028]_	Accuracy (SD)	ADI_accuracy_ score	B_U[0, 0.12]_	VWM (SD)
Experiment 5 (*n* = 24)	Positive	Neutral	914 (207)	-	-	0.90 (0.08)	-	-	0.86 (0.09)
		Positive	913 (203)	−1 (72)	0.24	0.89 (0.09)	2 (6)	0.51	0.85 (0.09)
		Threat	925 (199)	12 (49)	1.06	0.89 (0.08)	2 (5)	0.68	0.86 (0.08)
	Threat	Neutral	900 (205)	-	-	0.88 (0.11)	-	-	0.81 (0.10)
		Positive	912 (188)	12 (66)	0.96	0.88 (0.09)	0 (6)	0.11	0.82 (0.10)
		Threat	959 (237)	**60 (88)**	**54.07**	0.88 (0.09)	0 (5)	0.15	0.78 (0.11)

### VWM performance

A repeated measures ANOVA assessing the VWM change detection performance across all VWM and distractor conditions revealed a lower overall accuracy for threat-related spider stimuli in VWM, *F*(1,24) = 27.35, *p* <.001, *ƞ*^*2*^_*p*_ = 0.54. The difference across distractors was non-significant, *F*(2,48) = 2.50, *p* =.248, *ƞ*^*2*^_*p*_ = 0.06, however the interaction was significant, *F*(2,48) = 3.25, *p* =.048, *ƞ*^*2*^_*p*_ = 0.12.

Follow-up comparisons revealed that VWM change detection performance was significantly lower for threat-related VWM stimuli across all three distractor types, *t*(23) > 2.28, *p* <.032, *d*_*z*_ > 0.47. Further comparison of the difference between positive and threat-related VWM performance between distractors revealed that the decrement in performance was greater for the spider distractor versus the kitten, *t*(23) = 0.05, *p* =.010, *d*_*z*_ = 0.58, and marginally more relative to the bird, *t*(23) = 1.77, *p* =.091, *d*_*z*_ = 0.36. Thus, it appears that the spider distractor resulted in greater disruption to the spider VWM change detection task, beyond the generally lower performance for the spider VWM category. This is likely due to the visual similarity of the spider image selectively disrupting the representation of the different spider exemplar in VWM, versus the visually distinct kitten exemplar (Zhang & Peacock, 2025).

## Discussion

The results indicate that action preparation increased the interference from the VWM-matching threat-related distractors. In contrast to Experiment 4, this was limited only to the ADI_RT_ score comparison, rather than both ADI_accuracy_ and ADI_RT_ scores. The result therefore suggests that the interference seen in Experiment 4 reflected the combination of both temporal expectancy due to changes in trial structure, not just action preparation.

It should be noted that whilst the current task can measure interference from the briefly presented peripheral distractors, consistent with early attentional capture, it does not provide an index of spatial attentional capture. Thus, other cognitive process may be implicated in the interference (e.g., response selection). However, given the entirely task-irrelevant location of the distractor, any interference caused by the peripheral distractors must be contingent on a failure to inhibit these stimuli in attention.

Regardless of which factor had a more substantial effect on attention, the result indicates that enabling and encouraging action preparation increased the interference from task-irrelevant distractors which matched the contents of VWM. Importantly, this was the only aspect which differed from Experiment 1, where there was only weak anecdotal evidence favouring the null (i.e., B_H[0, 28]_ < 1).

Despite this clear evidence of VWM-driven capture by threat-related distractors, there was no evidence of increased capture by the positive distractor when matching the contents of VWM.

## Internal meta-analysis

To assess the overall magnitude of VWM-driven and VWM-independent attentional capture across the five behavioural studies, a Bayesian meta-analysis using the *brms* R package (Bürkner, 2017) was conducted, with model comparison via the *bridgesampling* package (Gronau et al., 2020). The analysis included Experiment as a random-effects factor to account for between-study heterogeneity. A normal prior distribution centered at zero with a standard deviation of 28ms was specified, reflecting the null hypothesis of no effect.

For interactions, Bayes factors were computed by comparing models that included the experimental conditions and their interaction against a null model containing only the experimental conditions without the interaction specified. For simpler contrasts between conditions, Bayes factors were computed be comparing models that included the experimental conditions against a null model containing only the intercept and random effects. All models were fit using Markov Chain Monte Carlo (MCMC) sampling with 4 chains of 5000 iterations each, discarding the first 50% as warmup and retaining the subsequent 2500 iterations per chain for posterior inference.

This analysis was limited to the primary reaction time dependent variable. The cumulative effects were computed for both key comparison of ADI scores between VWM-matching and mismatching conditions, as well as the ADI scores themselves within these VWM conditions.

### Cumulative threat distractor interference

**Fig. 8:**
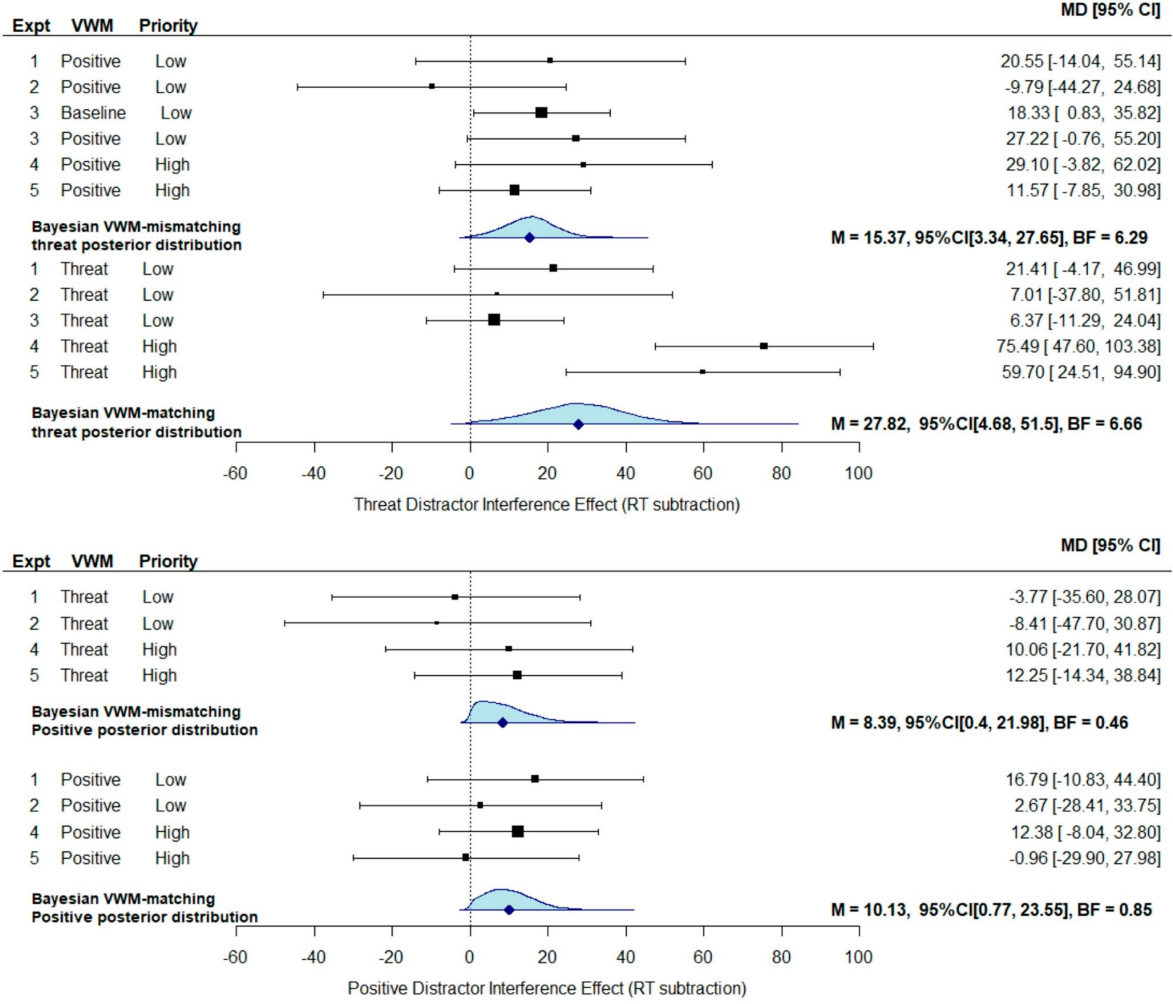
Forest plots depicting the individual fixed effects and cumulative posterior Bayesian estimate for both threat-related and positive affective distractor interference (ADI) reaction time (RT) scores, with 95% confidence intervals. Posterior estimates were computed using a half-normal prior centred on zero with a distribution of 28ms. ADI scores are presented for all experiments, visual working memory (VWM) types, and VWM task priority conditions. Bayes factors (BF) are reported for cumulative estimates

For the threat-related ADI_RT_ score, the inclusion of VWM match/mismatch as a moderator revealed a null effect with data showing anecdotal evidence, *M*_diff_ = 10.98, *SE*_*diff*_ = 6.38, 95% CI[-.91, 24.64], B_H[0, 28]_ = .97. Separate estimates showed substantial evidence that interference from the threat-related spider distractor was greater than the neutral bird distractor in both the VWM-match condition, M_diff_ = 27.82ms, *SE*_*diff*_ = 11.72, 95%CI[4.68, 51.50], B_H[0, 28]_ = 6.66, and the VWM-mismatch conditions, M_diff_ = 15.37ms, *SE*_*diff*_ = 6.17, 95%CI[3.34, 27.65], B_H[0, 28]_ = 6.29 (Fig. 8).

To test whether VWM prioritisation could account for the variation in VWM-matching ADI score, it was included as a moderator within the model alongside VWM match/mismatch. This revealed substantial evidence favouring an interaction between VWM-match/mismatch and VWM prioritisation, *M*_diff_ = 30.12, *SE*_*diff*_ = 13.48, 95% CI[5.43, 61.31], B_N[0,28]_ = 6.05, whereby experiments which encouraged prioritisation of the VWM representation (i.e., Experiments 4 and 5) resulted in greater ADI_RT_ score when matching the contents of VWM, versus mismatching, *M*_diff_ = 39.56, SE_diff_ = 12.91, 95% CI[13.63, 64.52], B_H[0,28]_ = 76.21. When, however, the task design did not encourage prioritisation of the VWM task (i.e., Experiments 1 – 3), the difference was in the opposing direction with VWM-matching interference being lower than in mismatching conditions, *M*_*diff*_ = −5.41ms, *SE*_*diff*_ = 4.49, 95CI[−16.56, − 0.16], B_H[0,28]_ = 0.21 and evidence substantially favouring the null.

### Cumulative positive distractor interference

The same comparison of VWM matching and mismatching ADI_RT_ scores for the positive distractors, however, revealed no significant difference, *M*_*diff*_ = 9.38ms, *SE*_*diff*_ = 6.67, 95CI[0.41, 25.05], B_H[0,28]_ = 0.49 (Fig. 8). Indeed, cumulative estimates across both VWM matching, *M*_*diff*_ = 10.13ms, *SE*_*diff*_ = 5.98, 95CI[0.77, 23.55], B_H[0,28]_ = 0.85, and VWM mismatching conditions, *M*_*diff*_ = 8.39ms, *SE*_*diff*_ = 5.89, 95CI[0.40, 21.98], B_H[0,28]_ = 0.46, revealed anecdotal evidence favouring the null hypothesis.

## General discussion

Established models of working memory and attention have posited that once a stimulus is prioritised in VWM it can induce an involuntary bias to task-irrelevant VWM-matching stimuli (Soto et al., 2008). The current investigation tested whether this top-down mechanism could induce distraction by affective stimuli presented in visual search tasks, and thus demonstrate that it could plausibly be an alternate driver of attentional biases to threat observed in real-world contexts. The data, however, revealed a more complex pattern of results than expected: Though there was cumulative evidence of modest value-driven interference from threat-related distractors independent of VWM, there was no substantial evidence that maintaining a representation of an affective category in VWM caused unconditional interference from exemplars matching the affective category. Only when task demands required that participants prioritise the VWM representation due to an unpredictable trial structure, or participants were able to prepare an action response specifically to the spider stimulus, did VWM-matching threat-related stimuli interfere more with visual search.

The evidence from Experiments 1–3 indicates that this VWM-driven capture is conditional, even for affective stimuli, and requires the features to be prioritised in VWM to cause attentional capture. The current result is, therefore, consistent with models which posit multiple stores or storage states in working memory, in which a stimulus representation can either be held in a prioritised “active” state or a less prioritised “accessory” state - with a representation only guiding attention when it is maintained in an active state (Houtkamp & Roelfsema, 2006; Olivers et al., 2011).

Though evidence suggests that, to some degree, multiple items can be maintained in an active state, this is strongly dependent upon current task demands requiring equal prioritisation of the stimuli (Beck & Vickery, 2019; Bahle et al., 2020). When, however, one stimulus is demarcated as more relevant, for instance through explicit cueing, this stimulus is promoted to a prioritised state where it guides attention, compared to the uncued stimulus which is demoted to an accessory state where it fails to influence selection (Dube et al., 2019; van Moorselaar et al., 2014).

Prioritisation in VWM is, however, not determined purely by explicit cueing and can be influenced by probabilistic temporal learning. For instance, when a target appears at a fixed timing, target matching irrelevant features do not guide attention until nearing the expected onset (Grubert & Eimer, 2018). Further, in a fixed interval VWM change detection task, participants are poorer at detecting a change from the VWM cue when a probe stimulus appears prior to the expected onset – suggesting a deprioritised state (Jin et al., 2020). In the current investigation, Experiment 4 demonstrates clearly that when the fixed temporal structure of a trial is made unpredictable, and both visual search and VWM change detection tasks are equally relevant at one time, then there is greater interference from VWM-matching distractors.

Temporal expectations are not, however, the only factor which determines the priority of a stimulus’ features in VWM. In Experiment 5, simply encouraging and allowing participants to prepare an action to the VWM change detection probe increased attentional capture by the memory matching peripheral distractors. In line with this finding, evidence has shown that action planning and preparation has a substantial effect on the prioritisation of stimuli in attention and working memory (for reviews see Heuer et al., 2020; Olivers & Roelfsema, 2020; van Ede, 2020). Whilst it could be argued that Experiments 1–3 still required participants to plan and prepare an action to the VWM change detection task, the preparation of this action plan was delayed by forcing it later into a sequence of actions by the response format. Stoet and Hommel (1999) have shown previously that the preparation of an action is disrupted if the same motor response is required for an earlier action sequence, as with Experiments 1–3, but not Experiments 4–5 where capture was found.

Thus, there appears to be distinction between the effect from unprepared action plans for prospective tasks and actively prepared actions. In support of this hypothesis, recent work by Trentin et al. (2024) has shown that when participants are able to prepare a specific motor response to a VWM change detection task prior to an intervening visual task then there is greater attentional capture by stimuli matching the contents of VWM, relative to when they are only provided with the relevant response later in the trials after the visual task, and are therefore unable to actively prepare the relevant response.

Memory-driven attentional capture in the current task design may have been especially dependent on this active prioritisation in VWM, relative to other tasks which show guidance from VWM when action preparation isn’t prioritised (e.g., Soto et al., 2005; Olivers et al., 2006). In these previous investigations the VWM-representation is usually a simple feature (e.g., coloured geometric shape) which is identical to a feature of the distractor. The current findings extend previous research by showing that VWM-driven capture extends to visually complex partially matching exemplars from the same category, but that this depends on the prioritised state in VWM. This is important, as in real-world situations the representation in VWM is unlikely to perfectively overlap with features in the environment, which dynamically vary depending on contextual factors such as lighting, orientation, and size.

### Boundary conditions of VWM-driven capture by affective categories

Given the extensive evidence that stimuli which match the contents of VWM typically capture attention across a range of tasks without manipulation of response preparation (Bahle et al., 2020; Beck et al., 2012; Downing., 2000; Olivers et al., 2006; Soto et al., 2005), it was surprising that there was substantial evidence against attentional capture in Experiments 1–3 when distractors were congruent with the contents of VWM (internal meta-analysisBF < 0.33). The current investigation differed from these prior investigations in two key ways, which will require further exploration in future research, specifically: spatial location of distractor stimuli and perceptual overlap between VWM contents and external distractors.

#### Spatial Location

As noted earlier, many of the earlier investigations present the irrelevant distractor in a potentially task-relevant location where a target will appear in the search array (Bahle et al., 2020; Beck et al., 2012; Downing., 2000; Olivers et al., 2006; Soto et al., 2005). Conversely, in the current investigation VWM-matching/mismatching distractors were presented in a consistent spatially irrelevant location. Though not tested in the current investigation, several previous studies have found that VWM-driven capture can be modulated by the spatial-relevance of the irrelevant distractor/cue. For instance, cueing which location a target will appear abolishes attentional capture by VWM-matching stimuli which appear in a different location (Pan & Soto, 2010). Similarly, presenting a VWM-matching shape in a consistent peripheral location rather than as an irrelevant dimension of a centrally presented secondary colour target also attenuates VWM-driven capture (Yang et al., 2024). This may have been further exaggerated in the current design with the brief stimulus presentation, which would have discouraged any allocation of spare attentional resources to peripheral locations.

Contingent capture research has found that goal-matching conceptually overlapping distractors capture attention even in peripheral spatially irrelevant locations (Wyble et al., 2013; Brown et al., 2020), these tasks utilise a single search goal which require the representation the goal features in a prioritised state, without balancing it alongside other target representations. The current findings, in combination with previous evidence that spatial location modulates VWM-driven attentional capture, suggest that VWM-driven capture by even affective categories isn’t automatic, and may only occur when the representation is in a more prioritised state. Surprisingly the interaction between spatial location and VWM-driven capture remains under explored with only a few studies testing this interaction. Future research is therefore required to explore this effect, and its potential interaction with VWM-representation priority.

#### VWM-distractor conceptual and perceptual overlap

Within many investigations showing a robust attentional capture by VWM-matching stimuli, there is complete overlap between the remembered stimulus and the external distractor. With the VWM representation and external distractor usually overlapping along simple colour or shape dimension (Bahle et al., 2020; Beck et al., 2012; Downing., 2000; Olivers et al., 2006; Soto et al., 2005). In the current investigation, however, the VWM cue and distractors were different images, and though they were from the same affective object category with shared features, they only partially overlapped.

Though previous studies have found that searching for conceptual categories can induced attentional capture by stimuli with partially overlapping features (Wyble et al., 2013; Brown et al., 2020), this has been when the conceptual goal is the only prioritised search goal and isn’t in an accessory state. It may be that for generalisation of attentional selection for partially overlapping distractors from the same category to occur, the VWM representation has to be in a prioritised state. As with spatial location, however, very little research has explored the generalisation of VWM-driven capture along conceptual or perceptual dimensions, or how this interacts with prioritisation. Future research is therefore needed to explore how these factors interact.

Alternatively, it could be that the current absence of VWM-driven capture may be due to participants utilising a different non-visual strategy, preventing overlap between internal representation and external distractors. Previous research has demonstrated that when participants are able to easily discriminate and label specific features of stimuli stored in working memory, they adopt a more verbal labelling strategy, reducing the representation of the stimulus as a visual template (Olivers et al., 2006). Though this strategy may be harder for abstract stimuli requiring the comparison of abstract shapes to shades of colour, exemplars from real-world conceptual categories may be easier to verbally label. The emphasis on the faster responding in Experiment 4 and 5 due to the advanced preparation of the VWM change detection response may have changed the strategy, as verbal/visual strategy can be influenced by various task demands (Brown & Wesley, 2013).

It should be noted that though threat-related distractors did capture attention in later tasks where VWM prioritisation was facilitated, VWM-matching positive distractors still did not capture attention. The influence of perceptual overlap between VWM and distractor may be a potential explanation for this. Though the exemplars of both kittens and spiders were from a similar range of perspectives, the small body and long legs of the spider had more consistent features across perspectives (i.e., more perspective invariant features; Biederman & Gerhardstein, 1993). Conversely, the kitten’s larger body and proportionally smaller legs would have had fewer overlapping features across perspectives, resulting in less frequent overlap across different exemplars (see Fig. 1).

This effect could have been further influenced by differences in familiarity with discriminating animal features (Amici et al., 2019), as the less familiar spider discrimination may have caused participants to rely on more salient or stereotypical features which would overlap more consistently across exemplars. Whilst for the kittens, more exemplar specific features may have been prioritised which did not overlap with distractor as frequently.

Regardless of the mechanism, the results suggest again, that VWM-driven capture is not due purely to conceptual or affective overlap, as merely thinking of an exemplar from a category was not sufficient to drive attention automatically to all other exemplars from that category. Instead, it likely requires the active maintenance of specific distractor-overlapping features in VWM.

Alongside the absence of VWM-driven capture by positive stimuli, there was also an absence of general goal-independent capture by positive kitten images versus neutral bird images, even when cumulative effects were meta-analytically computed, with Bayesian analysis anecdotally favouring the null. This was unexpected as prior evidence has shown that positive affective images can capture attention (see Pool et al., 2016 for review). In these previous investigations, however, these images are usually more reward associated or motivationally relevant (e.g., financial reward, erotic stimuli, facial expressions) rather than just positive or pleasant. From an evolutionary perspective, there may be less selective pressure to detect positive stimuli rapidly, relative to those that require a motivational response, especially threat where missing the stimulus can have fatal consequences. Indeed, evidence has suggested that motivationally rewarding stimuli have a greater effect on cognition than purely positive emotional stimuli (Chiew & Braver, 2014). The pleasant images of non-human infant animals may not have been sufficiently motivationally relevant or rewarding to capture attention, despite being perceived as positive.

### What does this tell us about goal-driven affective capture?

The current findings also question elements of the earlier *task-relevance* based model of top-down attentional bias to affective stimuli. This broad model suggests that the value-driven capture by affective stimuli is not unconditional and can be modulated by current top-down goals, but when individuals become aware of the affective dimension of the stimulus or it is relevant to a current task, then the automatic affective association influences attention (Schupp et al., 2000; Everaert et al., 2013, 2014).

Evidence for this theory comes from tasks in which the affective content of a stimulus is made task-relevant through explicit response instructions (e.g. categorising the affective content of the stimuli as positive/negative; Everaert et al., 2013, 2014; Hahn & Gronlund, 2007; Qiu et al., 2022, 2023; Stein et al., 2009; Van Dillen et al., 2011) or implicitly, by including the affective stimuli as part of the target set that much be detected (Lichtenstein-Vidne et al., 2012; Wirth & Wentura, 2023; Vromen et al., 2015, 2016). These previous investigations consistently reveal greater interference from threat-related stimuli when the affective content is currently relevant, compared to when it is task-irrelevant. Indeed, when the affective content is irrelevant, there can often be an absence of attentional capture by affective stimuli entirely.

The current studies, however, challenge assumptions of this theory, as (a) participants were aware of the affective stimuli and its content, and (b) it was directly relevant in the current paradigm, and yet there was no increase in attentional capture. The current results are therefore more in line with a *goal-driven* theory of capture by affective stimuli (e.g., Brown et al., 2020a; Brown, 2022), which proposes that top-down driven capture by emotion/value is mediated through established top-down mechanisms of attention (i.e., contingent capture; Folk et al., 1992), whereby the features associated with the object are prioritised as a search template (aka attentional set) to drive early selection. Indeed, the current studies reveal that top-down capture is heightened by an actively prioritised representation of a threat-related stimulus, rather than merely its relevance. Within this theory, the affective content of stimuli does not always bias attention directly, but instead the affective value biases attention indirectly through its representation as a goal – with individuals more likely to prioritise affective/valued outcomes as goals than neutral or low value outcomes.

The difference between goal-driven and relevance models of top-down attentional capture by threat have been relatively under-explored in the literature, with no studies designed specifically to differentiate them. The current findings which demonstrate VWM-driven capture by threat, independent of general awareness and relevance, do seem to favour a goal-driven mechanism. It is also consistent with previous findings revealing failures of task-relevance manipulations to increase capture by threat, which did not directly increase the priority of specific target-matching features as part of the goal. For instance, in studies utilising a verbal working memory change detection task, emotional words stored in working memory (e.g., fear, anger) failed to increase interference from emotional face distractors depicting that emotion in an intervening search task, despite their affective/semantic relevance (Grecucci et al., 2010; Moriya et al., 2014).

### Dual mechanisms of capture by threat

Alongside the increased attentional capture by VWM-matching threat-related stimuli, there was partial evidence that the threat-related distractor captured attention independent of the content so VWM, with a Bayesian meta-analysis revealing cumulative evidence for goal-independent capture. It should be noted, however, that across the individual studies goal-independent capture was not always consistent, with Bayes factors for the comparisons not always showing substantial evidence favouring capture (see Supplementary Materials 1 for similar results with frequentist statistics).

This is, however, not surprising as it is common that value-driven capture by spider stimuli in previous investigations is isolated to spider fearful individuals (Gerdes et al., 2008; Mogg & Bradley, 2006). Indeed, post hoc cumulative correlation between reported spider fear and the VWM-mismatch ADI score was significant (*k* = 5, *r* =.24, *p* =.004; see supplementary materials 3 for all individual differences analysis including anxiety) indicating that the effect was larger in spider fearful individuals. The current results that there was cumulative evidence in a non-selective sample is consistent with existing findings.

This evidence of goal-independent value-driven capture appears to contradict previous studies which find a lack of capture when participants pursue a competing non-threat-related goal (Brown et al., 2020a; Vogt et al., 2013; Vromen et al., 2016). A potential reason for the absence of attentional capture by goal-mismatching affective stimuli in these tasks may be due to differences in the type of goal manipulation. In the current investigation participants held the positive stimulus in VWM as a background goal, which would not require prioritisation during the presentation of the distractor. Conversely, previous tasks have often required participants to identify a briefly presented stimulus which could appear alongside the affective distractor (Brown et al., 2020a; Lichtenstein-Vidne et al., 2012; Vogt et al., 2013, 2022), or discriminate the non-affective features of a visually complex stimulus (Everaert et al., 2013; Stein et al., 2009).

These tasks would likely require more attentional prioritisation of the neutral/positive goal features at the point that the threat-related stimulus appears. It may be that only when the goal is sufficiently prioritised does it attenuate capture by threat. Consistent with this possibility, it has also been found that stimulus-driven attentional capture by goal-mismatching salient distractors still occurs when participants maintain goal-relevant features in VWM, but that this stimulus-driven capture is attenuated when the goal is currently prioritised as an active template (Plater et al., 2022).

Alternatively, it may be that previous investigations incidentally manipulated the perceptual load of the task. It is well established that when the perceptual demands of a task are high, due to a difficult perceptual discrimination or visually complex display, the interference from affective stimuli is attenuated (Doallo et al., 2006; Erthal et al., 2005; Fox et al., 2012; Yates et al., 2010). Thus, it may be that the previous target identification and discrimination tasks could have incidentally altered perceptual demands, resulting in an absence of goal-independent capture when the affective features weren’t relevant. The current visual search task, on the other hand, was based on the perceptually simple displays taken from long established investigations of perceptual load (Lavie, 1995; Forster & Lavie, 2008), meaning that there would have likely been sufficient capacity to process the affective value of the distractors.

Regardless of the reason that previous investigations only found interference from task-relevant threat, the current findings indicate that value-driven capture can occur alongside goal-driven capture. Such a dual mechanism would be highly beneficial and allow the flexibility to both monitor surrounding environments for potential threat even when irrelevant, as well as amplify processing of this threat automatically if it was perceived as currently relevant.

## Limitations

Within the current investigation, the sample sizes were based on power analyses derived from a previous investigation which found VWM-driven capture by peripheral task-irrelevant abstract shapes (Tan et al., 2015). It may be that more abstract or conceptual memory-driven capture may be a much smaller effect, requiring a larger sample to detect. Indeed, at the individual study level, Bayesian analyses suggested anecdotal evidence suggesting a lack of sensitivity. At the cumulative level though, the Bayesian meta-analysis did indicate substantial evidence favouring the null hypothesis in the comparison between VWM-matching versus mismatching distractors in low priority states, highlighting the consistency of the null effect. Future larger sample investigations will however be able to provide a more conclusive answer through further replication

As with many studies demonstrating attentional capture by VWM-matching stimuli (e.g., Olivers et al., 2006; van Moorselaar et al., 2014), the outcome measure was search speed and accuracy in detecting a target appearing concurrent to the distractor. Though this design allowed the test of distractor interference in direct competition with the target representation, it cannot determine the time-course of this interference. Whilst the stimuli were presented briefly and in an entirely task-irrelevant location, suggesting that an interference is due to early and involuntary attentional selection, this is not necessarily conclusive and interference could also occur at later stages (e.g., response selection). Future research aiming to isolate the specific mechanisms of value and goal-driven attentional biases over time should utilise continuous measures of attentional allocation such as event-related electrophysiology or eye-tracking, which can record the prolonged influence on later neural activity and dwell time. 

Within the current investigation, the affective categories of stimuli selected were positive kitten images and threat-related spider images. Whilst these stimuli have commonly been perceived as clearly positive and threat-related, with suggested evolutionary underpinnings for this association (Kringelbach et al., 2016; Rakinson & Derringer, 2008), and their affective content was confirmed in Samples as well as a larger independent sample (n = 82), the current effects may not generalise to all categories of affective stimuli. Future research should aim to replicate the current findings with a range of affective stimuli including emotional faces or Pavlovian conditioned affective stimuli. This would also isolate the purely value-driven elements from the associated visual features, which may be important to explore, as it has been suggested that some categories of affective stimuli are prioritised due to their low-level feature salience, not just their value associations (Calvo & Nummenmaa, 2008; Horstmann et al., 2012).

A final limitation is that the current findings were derived from samples of predominantly young UK-based female undergraduate students. Whilst there is no obvious confound in the result, due to the within-subjects design, for the current proposed mechanisms to be considered universal replication is required across different demographic groups. Additionally, any future individual differences research should aim to account for potential demographic factors moderating goal-driven and value-driven attentional biases. See Supplementary materials 3 for an exploratory analysis of individual differences in the current effects.

## Conclusions

The current findings point towards multiple mechanisms that cause the prioritisation of irrelevant threat-related stimuli in attention, rather than a single value-driven or goal-driven mechanism. Conventionally, these theories are framed as competing explanations for attentional capture by affective stimuli. There is, however, no clear reason that attentional capture cannot be caused independently by both these different mechanisms, though in different contexts. Based on the current findings it appears that affective stimuli may capture attention weakly independent of current goals in perceptually simple tasks, but when a concurrent goal to respond to affective stimuli is prioritised, the affective features which match this goal may be amplified in attention, causing further interference with ongoing tasks.

## Supplementary Information

Below is the link to the electronic supplementary material.

Supplementary Material 1 (DOCX 46.7 KB)

Supplementary Material 2 (DOCX 5.39 MB)

Supplementary Material 3 (DOCX 2.05 MB)

## Data Availability

All data and analyses scripts for the current investigation are available via the Open Science Framework (OSF link: https://osf.io/2ctjp/).

## Footnotes

1 This effect is also similar to a previous investigation that found evidence of attentional capture by infrequent threat-related peripheral stimuli in a near identical visual task (*d*_*z*_ = 0.90; Grimshaw et al., 2018). If photo-realistic affective stimuli were to cause attentional capture in the current paradigm, it would likely align with this effect.

2 Though frequentist power analyses are not directly compatible with Bayesian analyses, which were applied here, it is often observed that the p-value < 0.05 criterion of significance often aligns with a Bayes factor of 3, which is the criterion of moderate evidence (Dienes, 2014). Thus, the power analysis can provide a suitable approximation of the required sample to reach this level of evidence. To increase the suitability of the *a priori* power analysis, the standardised contrast used was taken from the same contrast used as the Bayesian prior (i.e., VWM-matching versus VWM-mismatching; Tan et al., 2015).
