# Supplementary material for: Exploring boundary conditions of goal-driven attentional capture by affective categories: the role of prioritisation in working memory
Source: Psychol Res. 2026 Feb 26;90(2):41. doi: 10.1007/s00426-025-02227-9 (PMC12945932; doi:10.1007/s00426-025-02227-9)
Supplement: Supplementary file 1 — Supplementary Material 1 (DOCX 46.7 KB) [file 426_2025_2227_MOESM1_ESM.docx]

**Supplementary materials 1: Frequentist Statistics**

**Statistical Analysis Overview**

In addition to the primary registered Bayesian analysis, a traditional frequentist analysis was also conducted to provide a complete overview of the data using ANOVA, and to facilitate accessibility those unfamiliar with Bayesian analyses. All pairwise comparisons were conducted using R software, and all ANOVAs were analysed using JASP software.

Across all five behavioural experiments, the key-preregistered contrasts using Affective Distractor Interference (ADI) scores were replicated with frequentist statistics. The ADI scores subtract out the when the distractor was neutral, thus isolating the effect of the purely affective content on visual search performance. For most experiments, the key contrast was between the ADI scores in the VWM-matching and VWM-mismatching conditions.

Following the key contrasts, and overall repeated measures ANOVA was conducted, which tested for general differences across all conditions which would not have been detected by the key pairwise contrasts (e.g., overall difference in performance. between positive and threat-related VWM types).

Finally, to assess whether there was any evidence of significant affective distractor interference in either the VWM matching or mismatching condition, the reaction time and accuracy when the distractor was neutral was contrasted with the positive or threat-related distractor conditions.

**Experiment 1a**

Experiment 1a was a 2 x 3 repeated measures design with Visual Working Memory stimulus type (positive kitten, threat-related spider) and Distractor type (neutral bird, positive kitten, threat-related spider) as the factors. For the overall ANOVA see S1 Table 1, for overall contrasts between neutral and affective distractors see S1 Table 2.

**Key hypothesised pair-wise contrasts**

The comparison of the threat-related spider ADI score between the positive and threat-related VWM conditions revealed non-significant differences, regardless of congruence with the VWM contents for both reaction time, *t*(21) = .04, *p* = .967, *d_z_* = .21, or accuracy based ADI score, *t*(21) = .60, *p* = .552, *d_z_* = .13.

The comparison of the positive kitten ADI score between the content matching positive VWM condition and mismatching threat-related VWM condition revealed no significant difference for either reaction time, *t*(21) = .97, *p* = .343, *d_z_* = .01, or accuracy based ADI measures, *t*(21) = -.31, *p* = .758, *d_z_* = .07.

| **Reaction time** | |
| --- | --- |
| VWM type | *F*(1,21) = 2.36, *p* = .140, *ƞ^2^_p_* = .10 |
| Distractor type | *F*(1,42) = 1.59, *p* = .216, *ƞ^2^_p_* = .07 |
| VWM type x Distractor type | *F*(1,42) = .68, *p* = .511, *ƞ^2^_p_* = .03 |
| **Accuracy** | |
| VWM type | *F*(1,21) = 6.19, *p* = .021, *ƞ^2^_p_* = .21 |
| Distractor type | *F*(1,42) = 1.11, *p* = .339, *ƞ^2^_p_* = .05 |
| VWM type x Distractor type | *F*(1,42) = .44, *p* = .650, *ƞ^2^_p_* = .02 |

S1 Table 1 Repeated measures ANOVA results for both visual search reaction time and accuracy dependent variables.

|  | VWM | Distractor | RT (SD) | RT DI score | t | p-value | Accuracy % (SD) | % DI score | t | p-value | VWM % (SD) |
| --- | --- | --- | --- | --- | --- | --- | --- | --- | --- | --- | --- |
| Experiment 1a (n = 22) | Positive | Neutral | 934 (176) | - | - | - | .83 (.08) | - | - | - | .83 (.08) |
|  |  | Positive | 950 (168) | 17 (66) | 1.19 | .247 | .80 (.08) | .01 (.04) | .66 | .514 | .79 (.08) |
|  |  | Threat | 954 (194) | 20 (83) | 1.16 | .257 | .81 (.10) | .01 (.05) | .61 | .551 | .81 (.10) |
|  | Threat | Neutral | 906 (173) | - | - | - | .82 (.11) | - | - | - | .82 (.11) |
|  |  | Positive | 903 (160) | -4 (76) | .23 | .819 | .79 (.12) | 0 (.05) | .22 | .830 | .79 (.12) |
|  |  | Threat | 928 (187) | 21 (61) | 1.64 | .116 | .80 (.10) | .02 (.07) | 1.19 | .249 | .80 (.10) |

S1 Table 2. Means and standard deviations for Reaction Time (RT) and accuracy (%), and Affective Distractor Interference (ADI) scores, as well as accuracy in the change detection task. Conventional statistics are reported (i.e., t-value, p-values) for all contrasts, all contrasts which are significant (*p* < .05) are highlighted in bold.

**Experiment 2**

Experiment 2 was a 2 x 3 repeated measures design with Visual Working Memory stimulus type (positive kitten, threat-related spider) and Distractor type (neutral bird, positive kitten, threat-related spider) as the factors. For the overall ANOVA see S1 Table 3, for overall contrasts between neutral and affective distractors see S1 Table 4.

**Key hypothesised pair-wise contrasts**

The comparison of the threat-related spider ADI score between the positive and threat-related VWM conditions revealed non-significant differences, regardless of congruence with the VWM contents for both reaction time, *t*(24) = .55, *p* = .585, *d_z_* = .11, or accuracy based ADI score, *t*(24) = .41, *p* = .683, *d_z_* = .08.

The comparison of the positive kitten ADI score between the content matching positive VWM condition and mismatching threat-related VWM condition revealed no significant difference for either reaction time, *t*(24) = .55, *p* = .585, *d_z_* = .11, or accuracy based ADI measures, *t*(24) = .13, *p* = .900, *d_z_* = -.03. To see the comparisons between neutral and affective distractors which make up the ADI scores, see S1 Table 2.

| **Reaction time** | |
| --- | --- |
| VWM type | *F*(1,24) = 1.91, *p* = .179, *ƞ^2^_p_* = .07 |
| Distractor type | *F*(1,48) = .03, *p* = .971, *ƞ^2^_p_* < .01 |
| VWM type x Distractor type | *F*(1,48) = .57, *p* = .568, *ƞ^2^_p_* = .02 |
| **Accuracy** | |
| VWM type | *F*(1,24) = 4.12, *p* = .054, *ƞ^2^_p_* = .15 |
| Distractor type | *F*(1,48) = 7.29, *p* = .002, *ƞ^2^_p_* = .23 |
| VWM type x Distractor type | *F*(1,48) = .10, *p* = .909, *ƞ^2^_p_* < .01 |

S1 Table 3. Repeated measures ANOVA results for both visual search reaction time and accuracy dependent variables.

|  | VWM | Distractor | RT (SD) | RT ADI score | t | p-value | Accuracy % (SD) | % ADI score | t | p-value | VWM % (SD) |
| --- | --- | --- | --- | --- | --- | --- | --- | --- | --- | --- | --- |
| Experiment 2 (n = 25) | Positive | Neutral | 929 (217) | - | - | - | .91 (.09) | - | - | - | .78 (.11) |
|  |  | Positive | 932 (208) | 3 (79) | .17 | .868 | .88 (.09) | .02 (.06) | 1.68 | .107 | .77 (.08) |
|  |  | Threat | 919 (190) | -10 (88) | .56 | .583 | .87 (.08) | **.04 (.07)** | **3.02** | **.006** | .78 (.08) |
|  | Threat | Neutral | 898 (167) | - |  | - | .89 (.07) | - | - | - | .77 (.10) |
|  |  | Positive | 890 (185) | -8 (100) | .42 | .678 | .87 (.10) | .02 (.11) | 1.10 | .281 | .76 (.09) |
|  |  | Threat | 905 (199) | 7 (114) | .31 | .762 | .84 (.11) | **.05 (.11)** | **2.42** | **.024** | .71 (.10) |

S1 Table 4. Means and standard deviations for Reaction Time (RT) and accuracy (%), and Affective Distractor Interference (ADI) scores, as well as accuracy in the change detection task. Conventional statistics are reported (i.e., t-value, p-values) for all contrasts, all contrasts which are significant (*p* < .05) are highlighted in bold.

**Experiment 3**

Experiment 3 was a 3 x 3 repeated measures design with VWM stimulus type (no VWM stimulus, positive kitten, threat-related spider) and Distractor type (no distractor, neutral bird, threat-related spider) as the factors. For the overall ANOVA see S1 Table 5, for overall contrasts between neutral and affective distractors see S1 Table 6.

**Key hypothesised pair-wise contrasts**

For Experiment 3, there were multiple specific contrasts designed to test different potential interpretations. These contrasts were conducted with general Distractor Interference (DI) scores, rather than ADI scores, which reflect the increase in disruption from any distractor type versus a no distractor baseline.

***Testing for VWM Content-Specific Effects on Distractor Interference***

Comparing the distractor present versus no distractor performance for the threat-related distractor revealed significant difference between threat VWM-matching and positive mismatching conditions for both reaction time, *t*(23) = .64, *p* =.530, *d_z_* = .13, and for accuracy, *t*(23) = 1.12, *p* =.275, *d_z_* = .23.

As a comparison, repeating this contrast between the positive kitten VWM and threat-related spider VWM conditions but with the neutral bird DI score revealed a similar pattern, with no significant differences for reaction time, *t*(23) = 1.47, *p* =.154, *d_z_* = .30, or accuracy, *t*(23) = .35, *p* =.730, *d_z_* = -.07.

The comparison between these two effects showed that the threat-related spider VWM task condition failed to substantially increase distractor interference for the spider distractor any more than the bird distractor, *t*(23) = 1.25, *p* =.223, *d_z_* = -.26. Indeed, purely numerically, the neutral bird distractor actually showed a larger increase in interference. The comparison with accuracy as the outcome measure revealed no significant difference, *t*(23) = 1.60, *p* =.124, *d_z_* = .32.

***Testing for General VWM Effects on Distractor Interference***

Pre-registered pairwise comparisons were also conducted to assess evidence for the general influence of VWM contents on processing of peripheral distractors. Average spider DI scores across both kitten and spider VWM conditions, versus spider DI scores in the no VWM task condition, revealed that there was no evidence of the VWM task increasing distractor interference on visual search reaction time, *t*(23) = .38, *p* =.708, *d_z_* = .08, and strong evidence that it had no effect on accuracy, *t*(23) = .34, *p* =.736, *d_z_* = -.07.

Similarly, there was no substantial evidence that the VWM task increased interference from neutral distractors, for reaction time, *t*(23) = .51, *p* =.614, *d_z_* = .10, and strong evidence that it had no effect on accuracy, *t*(23) = .26, *p* =.800, *d_z_* = .05.

***Testing for Purely VWM-Independent Affective Attentional Capture***

To further test whether the VWM change detection task influenced attentional capture by the animal flankers, the reaction time when the distractor was neutral and when it was threat-related were both compared to the baseline no distractor condition on blocks without the VWM task. The results are displayed in Table 4, and reveal that there was substantial evidence that the threat-related spider distractor disrupted visual search, conversely, there was no evidence suggesting attentional capture by the neutral bird distractor with anecdotal evidence favouring the null hypothesis. As can be seen from Table 4, the same comparisons with accuracy data were near zero and favoured the null hypothesis.

A follow-up unregistered analysis exploring the goal-independent ADI effects was conducted comparing the magnitude of the neutral DI score and threat-related DI score in the no VWM condition. This revealed a non-significant trend with the threat-related distractor causing marginally slower visual search versus the neutral distractor, *t*(23) = 2.05, *p* = .052, *d_z_* = .42, but not difference in accuracy, t(23) = .45, *p* = .656, *d_z_* = -.09.

Repeating this exploratory analysis, however, with all neutral versus threat-related DI scores collapsed across VWM conditions revealed a general significant value-driven ADI effect, (23) = 7.92, *p* = .023, *d_z_* = .50. There was no difference in accuracy for this comparison, t(23) = .11, *p* = .911, *d_z_* = -.02.

| **Reaction time** | |
| --- | --- |
| VWM type | *F*(2,46) = 39.92, *p* < .001, *ƞ^2^_p_* = .63 |
| Distractor type | *F*(2,46) = 6.06, *p* = .005, *ƞ^2^_p_* = .21 |
| VWM type x Distractor type | *F*(4,92) = 1.08, *p* = .369, *ƞ^2^_p_* = .05 |
| **Accuracy** | |
| VWM type | *F*(2,46) = .49, *p* = .616, *ƞ^2^_p_* = .02 |
| Distractor type | F*(*2,46) = .78, *p* = .465, *ƞ^2^_p_* = .03 |
| VWM type x Distractor type | *F*(4,92) = .75, *p* = .560, *ƞ^2^_p_* = .03 |

S1 Table 5. Repeated measures ANOVA results for both visual search reaction time and accuracy dependent variables.

|  | VWM | Distractor | RT (SD) | RT DI score | t | p-value | Accuracy % (SD) | % DI score | t | p-value | VWM % (SD) |
| --- | --- | --- | --- | --- | --- | --- | --- | --- | --- | --- | --- |
| Experiment 3 (n = 24) | No VWM | No distractor | 660 (112) | - | - | - | .91 (.06) | - | - | - | - |
|  |  | Neutral | 666 (111) | 6 (41) | .72 | .478 | .90 (.08) | .01 (.05) | 1.02 | .320 | - |
|  |  | Threat | 685 (127) | **24 (48)** | **2.47** | **.021** | .91 (.06) | .01 (.04) | .59 | .558 | - |
|  | Positive | No distractor | 863 (192) | - | - | - | .92 (.08) | - | - | - | .76 (.07) |
|  |  | Neutral | 856 (172) | -6 (61) | .50 | .619 | .91 (.08) | .01 (.05) | .59 | .564 | .81 (.08) |
|  |  | Threat | 884 (193) | 21 (82) | 1.25 | .224 | .90 (.08) | .02 (.06) | 1.43 | .167 | .80 (.07) |
|  | Threat | No distractor | 829 (146) | - | - | - | .90 (.08) | - | - | - | .83 (.09) |
|  |  | Neutral | 858 (172) | 29 (84) | 1.68 | .101 | .89 (.09) | .01 (.04) | 1.05 | .306 | .78 (.10) |
|  |  | Threat | 864 (168) | **35 (67)** | **2.58** | **.017** | .90 (.08) | < .01 (.05) | .11 | .910 | .80 (.11) |

S1 Table 6. Means and standard deviations for Reaction Time (RT) and accuracy (%), and Affective Distractor Interference (ADI) scores, as well as accuracy in the change detection task. Conventional statistics are reported (i.e., t-value, p-values) for all contrasts, all contrasts which are significant (*p* < .05) are highlighted in bold.

**Experiment 4**

Experiment 4 was a 2 x 3 repeated measures design with Visual Working Memory stimulus type (positive kitten, threat-related spider) and Distractor type (neutral bird, positive kitten, threat-related spider) as the factors. For the overall ANOVA see S1 Table 7, for overall contrasts between neutral and affective distractors see S1 Table 8.

**Key hypothesised pair-wise contrasts**

The comparison of the threat-related spider ADI score between the positive and threat-related VWM conditions revealed a non-significant difference, though there was a trend towards the threat-related ADI score being greater when matching the contents of the threat-related VWM, t(23) = 1.81, *p* = .083, *d_z_* = .37. The same comparison for accuracy, however, revealed a significant difference, with the VWM-matching condition resulting in a greater ADI score, *t*(23) = 4.29, *p* < .001, *d_z_* = .88.

The comparison of the positive kitten ADI score between the content matching positive VWM condition and mismatching threat-related VWM condition revealed no significant difference for either reaction time, t(23) = .12, *p* = .907, *d_z_* = .02, or accuracy based ADI measures, *t*(23) = .23, *p* = .824, *d_z_* = .05.

| **Reaction time** | |
| --- | --- |
| VWM type | *F*(1,23) = .87, *p* = .360, *ƞ^2^_p_* = .04 |
| Distractor type | *F*(1,46) = 14.84, *p* < .001, *ƞ^2^_p_* = .39 |
| VWM type x Distractor type | *F*(1,46) = 2.87, *p* = .067, *ƞ^2^_p_* = .11 |
| **Accuracy** | |
| VWM type | *F*(1,23) = .57, *p* = .458, *ƞ^2^_p_* = .02 |
| Distractor type | *F*(1,46) = 11.96, *p* < .001, *ƞ^2^_p_* = .34 |
| VWM type x Distractor type | *F*(1,46) = 14.13, *p* < .001, *ƞ^2^_p_* = .38 |

S1 Table 7. Repeated measures ANOVA results for both visual search reaction time and accuracy dependent variables.

|  | VWM | Distractor | RT (SD) | RT ADI score | t | p-value | Accuracy % (SD) | % ADI score | t | p-value |
| --- | --- | --- | --- | --- | --- | --- | --- | --- | --- | --- |
| Experiment 4  (n = 24) | Positive | Neutral | 932 (163) | - | - | - | .87 (.10) | - | - | - |
|  |  | Positive | 944 (161) | 12 (51) | 1.19 | .247 | .88 (.10) | 0 (.07) | .12 | .906 |
|  |  | Threat | 961 (185) | 29 (82) | 1.73 | .097 | .87 (.09) | 0 (.08) | .05 | .957 |
|  | Threat | Neutral | 934 (159) | - | - | - | .89 (.07) | - | - | - |
|  |  | Positive | 944 (200) | 10 (79) | .62 | .541 | .90 (.08) | -.01 (.05) | .46 | .651 |
|  |  | Threat | 1009 (192) | 75 (70) | **5.31** | **< .001** | .81 (.12) | .08 (.07) | **6.04** | **<.001** |

S1 Table 8. Means and standard deviations for Reaction Time (RT) and accuracy (%), and Affective Distractor Interference (ADI) scores, as well as accuracy in the change detection task. Conventional statistics are reported (i.e., t-value, p-values) for all contrasts, all contrasts which are significant (*p* < .05) are highlighted in bold.

**Experiment 5**

Experiment 5 was a 2 x 3 repeated measures design with Visual Working Memory stimulus type (positive kitten, threat-related spider) and Distractor type (neutral bird, positive kitten, threat-related spider) as the factors. For the overall ANOVA see S1 Table 9, for overall contrasts between neutral and affective distractors see S1 Table 10.

**Key hypothesised pair-wise contrasts**

The comparison of the threat-related spider ADI score between the positive and threat-related VWM conditions revealed a significant effect, with the threat-related ADI score being greater when matching the contents of VWM, *t*(23) = 2.39, *p* = .026, *d_z_* = .49. The same comparison for accuracy data revealed a non-significant difference, *t*(23) = .83, *p* = .416, *d_z_* = .17.

The comparison of the positive kitten ADI score between the content matching positive VWM condition and mismatching threat-related VWM condition revealed no significant difference for either reaction time, *t*(23) = .69, *p* = .499, *d_z_* = -14, or accuracy based ADI measures, *t*(23) = 1.03, *p* = .316, *d_z_* = .21.

| **Reaction time** | |
| --- | --- |
| VWM type | *F*(1,23) = .11, *p* = .749, *ƞ^2^_p_* = .01 |
| Distractor type | *F*(1,46) = 5.67, *p* = .006, *ƞ^2^_p_* = .20 |
| VWM type x Distractor type | *F*(1,46) = 3.03, *p* = .058, *ƞ^2^_p_* = .12 |
| **Accuracy** | |
| Main effect of VWM | *F*(1,23) = 2.67, *p* = .116, *ƞ^2^_p_* = .10 |
| Main effect of Distractor type | *F*(1,46) = .98, *p* = .382, *ƞ^2^_p_* = .04 |
| VWM type x Distractor type | *F*(1,46) = .60, *p* = .551 , *ƞ^2^_p_* = .02 |

S1 Table 9. Repeated measures ANOVA results for both visual search reaction time and accuracy dependent variables.

|  | VWM | Distractor | RT (SD) | RT ADI score | t | p-value | Accuracy % (SD) | % ADI score | t | p-value | VWM % (SD) |
| --- | --- | --- | --- | --- | --- | --- | --- | --- | --- | --- | --- |
| Experiment 5 (n = 24) | Positive | Neutral | 914 (207) | - | - | - | .90 (.08) | - | - | - | .86 (.09) |
|  |  | Positive | 913 (203) | -1 (72) | .06 | .949 | .89 (.09) | 2 (6) | 1.31 | .203 | .85 (.09) |
|  |  | Threat | 925 (199) | 12 (49) | 1.17 | .255 | .89 (.08) | 2 (5) | 1.62 | .120 | .86 (.08) |
|  | Threat | Neutral | 900 (205) | - | - | - | .88 (.11) | - | - | - | .81 (.10) |
|  |  | Positive | 912 (188) | 12 (66) | .90 | .376 | .88 (.09) | 0 (6) | -.14 | .89 | .82 (.10) |
|  |  | Threat | 959 (237) | **60 (88)** | **3.32** | **.003** | .88 (.09) | 0 (5) | .44 | .66 | .78 (.11) |

S1 Table 10. Means and standard deviations for Reaction Time (RT) and accuracy (%), and Affective Distractor Interference (ADI) scores, as well as accuracy in the change detection task. Conventional statistics are reported (i.e., t-value, p-values) for all contrasts, all contrasts which are significant (*p* < .05) are highlighted in bold.
