# Supplementary material for: Exploring boundary conditions of goal-driven attentional capture by affective categories: the role of prioritisation in working memory
Source: Psychol Res. 2026 Feb 26;90(2):41. doi: 10.1007/s00426-025-02227-9 (PMC12945932; doi:10.1007/s00426-025-02227-9)
Supplement: Supplementary file 2 — Supplementary Material 2 (DOCX 5.39 MB) [file 426_2025_2227_MOESM2_ESM.docx]

**Supplementary Materials 2: Stimulus Affective Ratings**

**Meta-analysis of Affective Ratings Across Behavioural Experiments**

Across all behavioural studies within the current investigation (*k* = 5; total *N* = 119), participants provided valence and arousal ratings for the distractor stimuli (see Experiment 1a methods for full details). To determine whether the stimuli were perceived as sufficiently affective and emotionally arousing, in these samples specifically, an exploratory fixed effects meta-analysis was conducted using the *metafor* package (Viechtbauer, 2010). This allowed both the assessment of the cumulative difference across studies, as well as whether there was significant heterogeneity in the ratings across studies (see S2 Figure 1 for forest plots).


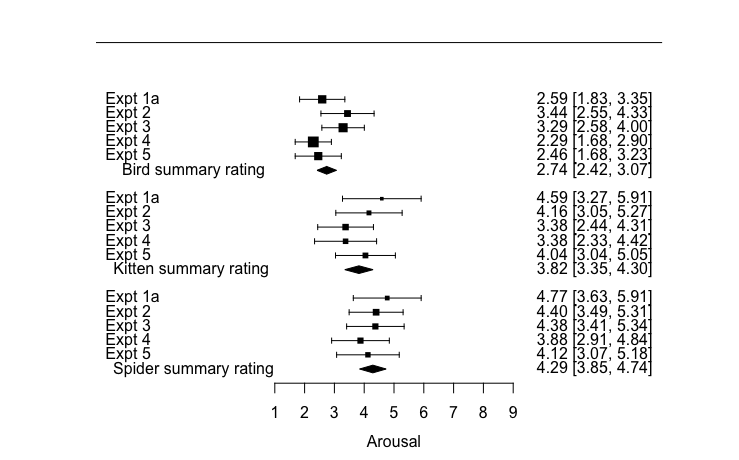

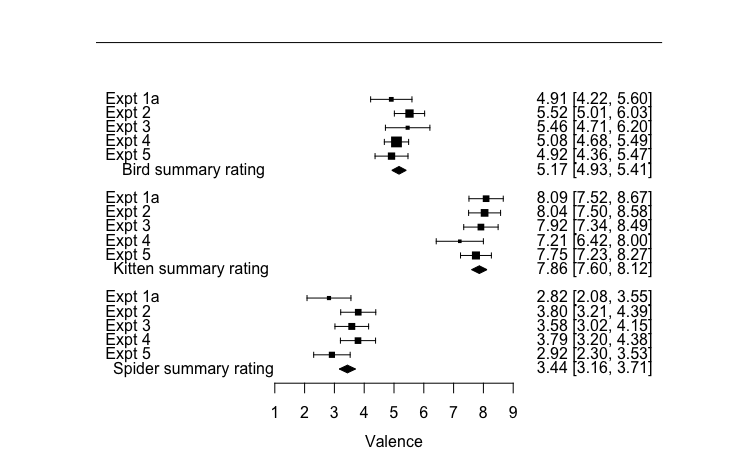


S2 Figure 1. Forest plots depicting the cumulative fixed effects for both valence and arousal scores across the behavioural experiments, with 95% confidence intervals.

For valence, the overall ratings were significantly different across distractor types, *Q_M_*(2) = 675.48, *p* < .001. With the positive kitten distractors *M* = 7.86, *SE* = .13, being significantly more positive versus the neutral bird distractor, *M* = 5.17, *SE* = .12, *p* < .001, 95CI[2.33, 3.05]. The threat-related spider distractor, *M* = 3.44, *SE* = .14, were also significantly more unpleasant than the neutral bird distractor, *p* < .001, 95CI[-2.11, -1.37]. There was no significant evidence of heterogeneity across all effects, *Q*(5) = 8.60, *p >* .072.

The analysis of arousal ratings also showed the same expected differences, with both the positive kitten stimuli, *M* = 3.82, *SE* = .24, and spider distractor stimuli, *M* = 4.29, *SE* = .23, being significantly more arousing relative to the neutral bird stimuli, *M* = 2.74, *SE* = .17, *p* < .001, 95CI[.48, 1.64]. Importantly, heterogeneity was non-significant for all effects, *Q*(5) = 7.42, *p* > .115, indicating a relatively consistent judgment of affective arousal across studies, making variation in subsequent studies unlikely to be due to differences in the affective content of the distractors.

**Independent Sample Ratings**

***Participants***

An initial sample of 128 participants access the link to the online study which was advertised on social media, though 46 were excluded for providing incomplete data. The final sample of 82 participants consisted of 16 men and 66 women, the mean age of which was 23.80 years *SD* = 4.99. The average level of trait anxiety was 46.8, *SD* = 9.64, and state anxiety was 36.72, *SD* = 9.81. The average spider fear score was, M = 2.76, SD = 1.93.

***Stimuli and Procedure***

Participants completed the STAI and FSQ, and self-assessment manikin image rating task, which were completed remotely at home using Qualtrics software.

**Results**

Three-way repeated measures ANOVA’s on valence and arousal ratings revealed a significant overall difference across the stimulus categories, Valence: *F*(1.82, 147.41) = 65.84, *p* < .001, *ƞ^2^_p_* = .45 (Huynh-Feldt corrected); Arousal: *F*(1.70, 137.62) = 9.52, *p* < .001, *ƞ^2^_p_* = .11. Follow-up paired t-tests between the affective and neutral stimuli confirmed that both the kitten and spider affective stimuli were perceived as more positive/negative and more emotionally arousing relative to the neutral bird image (see S2 Table 1).

|  |  | Mean Valence Rating (SD) | Cohen’s *d_z_* | p-value | Mean Arousal Ratings (SD) | Cohen’s *d_z_* | p-value |
| --- | --- | --- | --- | --- | --- | --- | --- |
| Cumulative effects from Experiments 1 – 5  (N = 119) | Neutral bird stimuli | 5.17 (1.35) | - | - | 2.74 (1.82) | - | - |
|  | Positive kitten stimuli | 7.86 (1.45) | 1.34 | < .001 | 3.82 (2.64) | .33 | < .001 |
|  | Threat spider stimuli | 3.44 (1.52) | -.85 | < .001 | 4.29 (2.48) | .49 | < .001 |
| Independent sample (n = 82) | Neutral bird stimuli | 3.66 (2) | - | - | 2.90 (1.99) | - | - |
|  | Positive kitten stimuli | 6.93 (2.23) | 1.03 | .001 | 4.63 (2.70) | .52 | .001 |
|  | Threat spider stimuli | 2.90 (2.52) | -.26 | .024 | 4.02 (3.02) | .36 | .002 |

S2 Table 1. Mean and standard deviation (SD) ratings from the self-assessment manikin along dimensions of valence and arousal for all peripheral distractor stimuli. Effect sizes and significance values from two-tailed t-tests are reported for the comparison versus neutral baseline distractor stimuli.
