# Supplementary material for: Exploring boundary conditions of goal-driven attentional capture by affective categories: the role of prioritisation in working memory
Source: Psychol Res. 2026 Feb 26;90(2):41. doi: 10.1007/s00426-025-02227-9 (PMC12945932; doi:10.1007/s00426-025-02227-9)
Supplement: Supplementary file 3 — Supplementary Material 3 (DOCX 2.05 MB) [file 426_2025_2227_MOESM3_ESM.docx]

**Supplementary Materials 3: Exploratory Individual Differences Analysis**

In addition to the primary within-subjects analyses, self-reported state and trait anxiety, and spider fear were also recorded. To assess whether there was any relationship between these measures and affective distractor interference across studies, a fixed effects meta-analysis was conducted for the three measures and ADI scores in both VWM matching and mismatching conditions. These meta-correlations were Bonferroni corrected for the six analyses (corrected ⍺ = .008). To caveat this analysis the current samples were not sufficiently powered to reliably detect individual differences, which can result in biased cumulative estimates (Lin, 2018). These should therefore be interpreted with caution, but were conducted to facilitate future individual differences research.


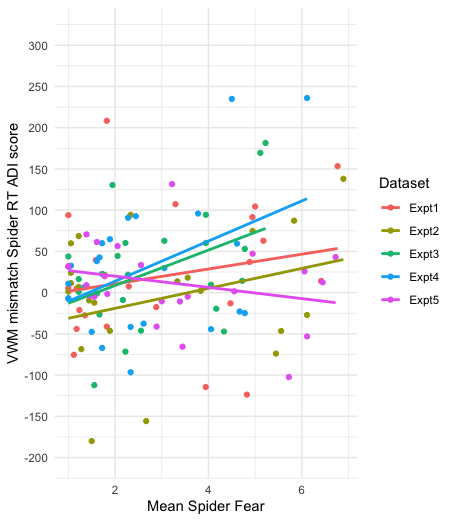

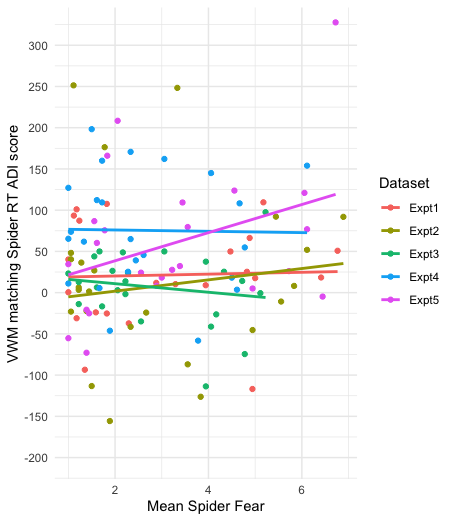


S3 Figure 1. Scatterplots depicting all relationships between the mean Fear of Spider score and spider Reaction Time (RT) Affective Distractor Interference (ADI) score, in both Visual Working Memory (VWM) matching and mismatching conditions. A colour version is available online.

The analysis revealed that there was a significant positive correlation between self-reported spider fear and the threat-related spider ADI score in the VWM-mismatch condition, *r* = .24, *p* = .004, 95CI[.08, .40], but not in the VWM-matching condition, *r* = .08, *p* = .349, 95CI[-.09, .26]. The spider unrelated factors of trait and state anxiety were not significantly correlated with the threat-related spider ADI score in either VWM-matching or mismatching condition, *r* < .07, *p* > .421.
